# Proteomic Atlas of Atherosclerosis: The Contribution of Proteoglycans to Sex Differences, Plaque Phenotypes, and Outcomes

**DOI:** 10.1161/CIRCRESAHA.123.322590

**Published:** 2023-08-30

**Authors:** Konstantinos Theofilatos, Stefan Stojkovic, Maria Hasman, Sander W. van der Laan, Ferheen Baig, Javier Barallobre-Barreiro, Lukas Emanuel Schmidt, Siqi Yin, Xiaoke Yin, Sean Burnap, Bhawana Singh, Jude Popham, Olesya Harkot, Stephanie Kampf, Maja Carina Nackenhorst, Andreas Strassl, Christian Loewe, Svitlana Demyanets, Christoph Neumayer, Martin Bilban, Christian Hengstenberg, Kurt Huber, Gerard Pasterkamp, Johann Wojta, Manuel Mayr

**Affiliations:** King’s British Heart Foundation Centre, Kings College London, United Kingdom (K.T., M.H., F.B., J.B.B., L.E.S., S.Y., X.Y., S.B., B.S., J.P., M.M.).; Division of Cardiology, Department of Internal Medicine II (S.S., O.H., C.H., J.W., M.M.), Medical University of Vienna, Austria.; Division of Vascular Surgery, Department of Surgery (S.K., C.N.), Medical University of Vienna, Austria.; Department of Pathology (M.C.N.), Medical University of Vienna, Austria.; Division of Cardiovascular and Interventional Radiology, Department of Biomedical Imaging and Image-Guided Therapy (A.S., C.L.), Medical University of Vienna, Austria.; Department of Laboratory Medicine (S.D.), Medical University of Vienna, Austria.; Core Facilities (M.B.), Medical University of Vienna, Austria.; Central Diagnostics Laboratory, Division Laboratories, Pharmacy, and Biomedical Genetics, University Medical Center Utrecht, Utrecht University, the Netherlands (S.W.v.d.L., G.P.).; Third Medical Department, Wilhelminenspital, and Sigmund Freud University, Medical Faculty, Vienna, Austria (K.H.).; Ludwig Boltzmann Institute for Cardiovascular Research, Vienna, Austria (J.W.).

**Keywords:** atherosclerosis, inflammation, machine learning, neutrophils, proteoglycans, proteomics, smooth muscle cells

## Abstract

**BACKGROUND:**

Using proteomics, we aimed to reveal molecular types of human atherosclerotic lesions and study their associations with histology, imaging, and cardiovascular outcomes.

**METHODS:**

Two hundred nineteen carotid endarterectomy samples were procured from 120 patients. A sequential protein extraction protocol was employed in conjunction with multiplexed, discovery proteomics. To focus on extracellular proteins, parallel reaction monitoring was employed for targeted proteomics. Proteomic signatures were integrated with bulk, single-cell, and spatial RNA-sequencing data, and validated in 200 patients from the Athero-Express Biobank study.

**RESULTS:**

This extensive proteomics analysis identified plaque inflammation and calcification signatures, which were inversely correlated and validated using targeted proteomics. The inflammation signature was characterized by the presence of neutrophil-derived proteins, such as S100A8/9 (calprotectin) and myeloperoxidase, whereas the calcification signature included fetuin-A, osteopontin, and gamma-carboxylated proteins. The proteomics data also revealed sex differences in atherosclerosis, with large-aggregating proteoglycans versican and aggrecan being more abundant in females and exhibiting an inverse correlation with estradiol levels. The integration of RNA-sequencing data attributed the inflammation signature predominantly to neutrophils and macrophages, and the calcification and sex signatures to smooth muscle cells, except for certain plasma proteins that were not expressed but retained in plaques, such as fetuin-A. Dimensionality reduction and machine learning techniques were applied to identify 4 distinct plaque phenotypes based on proteomics data. A protein signature of 4 key proteins (calponin, protein C, serpin H1, and versican) predicted future cardiovascular mortality with an area under the curve of 75% and 67.5% in the discovery and validation cohort, respectively, surpassing the prognostic performance of imaging and histology.

**CONCLUSIONS:**

Plaque proteomics redefined clinically relevant patient groups with distinct outcomes, identifying subgroups of male and female patients with elevated risk of future cardiovascular events.

**Novelty and Significance**

**What Is Known?**

- Atherosclerotic plaques are currently characterized using imaging and histology.
- Transcriptomic signatures have been associated with plaque subtypes.

**What New Information Does This Article ­Contribute?**

- This proteomics study doubles the coverage of the plaque proteome in large cohorts.
- Calcification and inflammatory protein signatures of atherosclerosis were identified and integrated with bulk, single-cell, and spatial transcriptomics.
- A sex-specific signature, enriched in large-aggregating proteoglycans, contributed to grouping patients into clusters with different outcome trajectories.

We generated the largest proteomics data set of fresh-frozen human plaques to date, combining untargeted and targeted proteomics with network reconstruction and clustering techniques to gain molecular insights into protein changes related to clinical characteristics. The proteomic signatures of plaque composition were found to predict long-term, adverse cardiovascular events, improving risk stratification for patients with advanced atherosclerosis. Notably, the proteoglycan content of plaques was inversely associated with serum estradiol levels and contributed to the stratification of predominantly female patients into a subgroup associated with worse cardiovascular outcomes.

**In This Issue, see p 539**

**Meet the First Author, see p 540**

Despite decades of research, there remain ambiguities on the mechanisms that induce atherosclerotic plaque instability. According to the response-to-retention hypothesis,^1^ the binding of cholesterol-containing lipoprotein particles to intimal proteoglycans is the central pathogenic process in early atherogenesis. Once retained, lipoproteins get oxidized, accumulate in foam cells, and provoke a cascade of inflammatory processes that drive the formation of atherosclerosis. In addition to its critical role in the retention of lipoprotein particles, ECM (extracellular matrix) degradation processes are causally linked to the development of vulnerable plaques.^2^ The ECM, in particular of the fibrous cap, ultimately defines the propensity of plaques to rupture leading to atherothrombotic events. There is a need to further understand how ECM composition differs across plaque phenotypes. The matrisome refers to the ensemble of ECM and ECM-associated proteins.^3^ Proteomics offers an opportunity to define the protein composition of human atherosclerosis that can advance current assessments by histology and imaging. Till now, mapping initiatives lack information on large blood vessels and atherosclerotic plaques.

According to the modified American Heart Association classification, there are 8 types of atherosclerotic lesions: intimal thickening, fibroatheroma, late fibroatheroma, healed plaque rupture, fibrocalcific plaques, erosions, thin-capped atheroma, and ruptured plaques.^4^ Calcification is supposedly a late feature of advanced atherosclerotic lesions.^5^ According to ultrasound measurements, atherosclerotic plaques are classified as echolucent, mixed, or echogenic. Echolucent plaques are soft, unstable plaques with a thin fibrous cap, a necrotic lipid core, and inflammation. Echolucent plaques are more prone to rupture and cause clinically overt disease.^6^ In contrast, echogenic plaques are less likely to cause clinical symptoms due to reduced inflammation and increased ECM content.^7^

Until now, attempts to molecularly phenotype atherosclerotic plaques have primarily relied on transcriptomics.^8^ We developed novel mass spectrometry (MS)–based methods to characterize extracellular proteins.^9,10^ We successfully applied these methods to a small cohort of atherosclerotic plaques from patients with symptomatic and asymptomatic carotid stenosis, revealing a molecular signature with known and novel inflammatory biomarkers that could be measured in plasma and predicted future cardiovascular events.^11^ Combining large-scale tissue proteomics with bioinformatics, we strove to reveal molecular types of human atherosclerotic lesions, study their associations with the current classifications by histology and imaging techniques and relate them to cardiovascular outcomes in 2 independent cohorts.

## METHODS

### Data Availability

Summary data and additional analyses that support the findings of this study are available from the corresponding authors on reasonable request. Proteomics and spatial transcriptomics data were deposited at the Proteomics Identifications (PRIDE) and Gene Expression Omnibus (GEO) repositories, respectively.

### Patient Cohorts

One hundred twenty patients with carotid artery stenosis undergoing carotid endarterectomy were included in the discovery cohort from Medical University of Vienna, Austria. The validation cohort consisted of 200 patients undergoing carotid endarterectomy from the Athero-Express Biobank study.^12^

### Proteomics Analysis by MS

Proteins were extracted using sequential incubation with 0.5 M sodium chloride (NaCl), 0.1% sodium dodecyl sulfate (SDS), and 4 M guanidine hydrochloride (GuHCl).^10^ All extracts were labeled using tandem mass tags and analyzed on an Orbitrap Fusion Lumos Tribrid MS for proteomics (Thermo Scientific). A parallel reaction monitoring method was developed on a Q Exactive HF MS (Thermo Scientific). For validation in the Athero-Express Biobank,^12^ proteins were extracted using 0.5 M NaCl and 4M GuHCl, followed by label-free quantitation by MS in the GuHCl extracts.

### Enzyme-Linked Immunosorbent Assay

ELISA measurements were conducted in plasma samples of the discovery cohort using DuoSet ELISA Ancillary Reagent Kit 2 (DY008B).

### RNA-Sequencing Analysis

Publicly available single-cell RNA-sequencing (scRNAseq) data sets of carotid endarterectomies from 12 patients were integrated using the Harmony tool.^13^ The spatial RNA-seq analysis was conducted on sections from 2 asymptomatic, male patients with 80% to 90% stenosis.

### Statistical and Bioinformatics Analysis

The proteins identified in the GuHCl and NaCl extracts were filtered for ECM and extracellular proteins. The data set was further filtered for missing values. The limma package was used to compare different phenotypes using the Ebayes algorithm correcting for selected covariates. For discovery proteomics nominal *P* values were used to identify dysregulated proteins. *P* values corrected for multiple testing are provided in the Supplemental Material. For validation in the Athero-Express cohort, correlation, and enrichment analyses, significance was based on Benjamini-Hochberg corrected *P* values. Proteins belonging to the revealed proteomics signatures were considered potential inputs to classification models to predict the primary end points. An ensemble dimensionality reduction technique was used deploying a multiobjective evolutionary algorithm to identify the optimal feature subset to be used as input to a support vector machines model and optimize the support vector machine’s parameter values. A more detailed methods description is provided in the Supplemental Material.

## RESULTS

### Expanding the Coverage of the Human Plaque Proteome

Our discovery cohort consisted of 219 human carotid endarterectomy specimens from 120 patients (Table S1). No statistically significant differences were observed between symptomatic and asymptomatic patients based on ultrasound or histology plaque classification. We validated our findings using carotid plaques from 200 patients from the Athero-Express Biobank.^12^ Statistical comparisons were corrected for statin use, which was higher in symptomatic patients. With proteomics data collected from 419 carotid endarterectomy specimens, this study surpasses previous carotid plaque proteomics studies^11,14–19^ (Figure S1). Quantitative, multiplexed proteomics with tandem mass tags (TMT) was conducted on 3 extracts resulting in consistent quantification of 1459 predominantly cellular proteins in SDS (Summary Proteomics and Statistics Results of TMT Proteomics Data on SDS Extract in the Supplemental Material) and 381 extracellular proteins in NaCl and GuHCl fractions (283 soluble and 286 core matrisome proteins; Summary results and Statistics of the TMT Proteomics Using the NaCl Extract and Summary results and Statistics of the TMT Proteomics Using the GuHCl Extract in the Supplemental Material).

### Distinct Cellular Proteome Characteristics of the Plaque Core and Periphery

**Figure 1.**
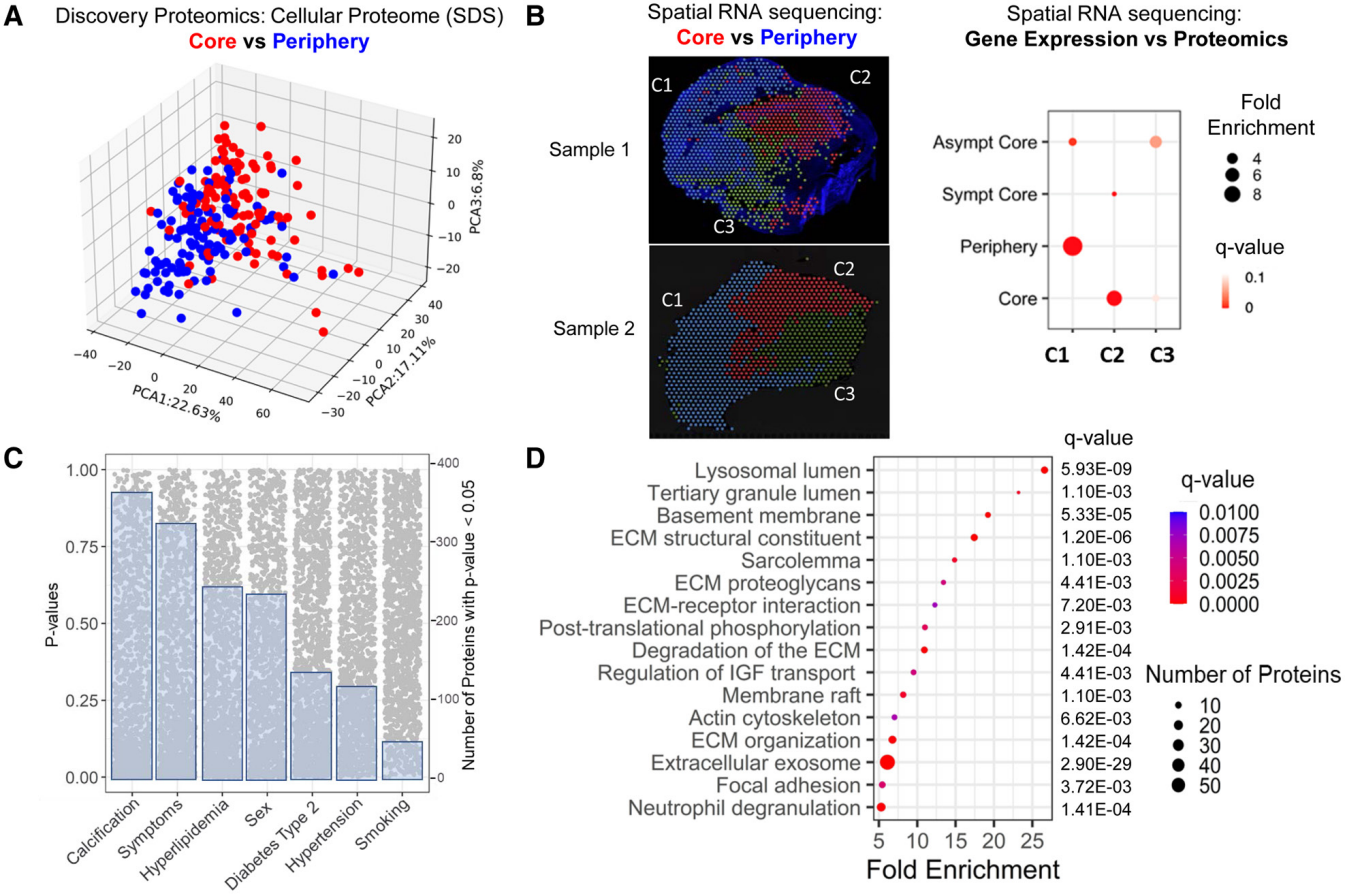
**Proteomic signatures of the plaque core and periphery. A**, Principal component analysis (PCA) visualization of the carotid plaque samples from both the core (red) and periphery (blue) of the plaque using the cellular proteome (sodium dodecyl sulfate [SDS] extract). **B**, Spatial RNA-sequencing (RNAseq): Three regional clusters (C1, C2, and C3) were revealed from spatial RNAseq of 2 carotid plaques. C1 and C2 corresponded to the protein changes identified in plaque periphery (blue) and core samples (red; Fisher exact test *Q*-value <0.05) and C3 corresponds to an intermediate position. There was agreement between proteomic and spatial RNA signatures (C1 vs C2) with Benjamini-Hochberg–corrected *Q* values for asymptomatic (asympt) cores vs C1: 1.98×10^-5^, asymptomatic core vs C3: 0.048, symptomatic (sympt) core vs C2: 8.84×10^-^^4^, periphery vs C1: 2.24×10^-^^26^, core vs C2: 4.58×10^-^^19^. Linear color scale was used for *Q* values. **C**, Changes in the SDS extracts of the plaque cores according to cardiovascular risk factors, sex, symptoms, and calcification. Differential protein analysis was conducted using the Ebayes method of the limma package adjusted for age and sex. Scatterplots show the nominal *P* values. Blue boxes depict the number of proteins with nominal *P*<0.05 in each comparison (out of 1459 consistently quantified proteins in the SDS fraction). **D**, Pathway enrichment analysis of significantly dysregulated proteins (SDS: core vs periphery). The total quantified proteins were used as background in the enrichment analysis. Linear color scale was used for *Q* values. ECM indicates extracellular matrix. IGF indicates insulin-like growth factor.

The carotid endarterectomies from the discovery cohort were divided into peripheral and core specimens. The proteomics analysis of cellular protein extracts (SDS) differentiated core from periphery of the plaques (Figure 1A). Spatial RNAseq data (Differential Expression Results Comparing Clusters of Spatial RNASeq Data From Carotid Plaques in the Supplemental Material) supported the findings on regional differences. Enrichment analysis of proteomics and spatial transcriptomics data revealed 3 positional clusters: cluster 1 representing changes in the periphery, cluster 2 in the core, and cluster 3 corresponding to an intermediate area (Figure 1B). Changes in cellular protein content in plaque cores were associated with clinical characteristics, particularly calcification, symptomatic status, hyperlipidemia, and sex (Figure 1C). The significant changes in the SDS fraction were primarily related to lysosomal degradation, ECM, and ECM-associated proteins (Figure 1D).

Quantification of cell markers for smooth muscle cells (SMC), macrophages, and platelets revealed strong positive correlations between SMC markers (ACTA [aortic smooth muscle actin], TAGL [transgelin], CNN1 [calponin-1], CALD1 [caldesmon]) and structural ECM proteins (Figure S2), while CD14 (monocyte differentiation antigen CD14) and CD45 (PTPRC [receptor-type tyrosine-protein phosphatase C]) showed positive correlations with inflammation-related proteins and an inverse relationship to structural ECM proteins, especially collagens. In contrast, alternative macrophage markers such as C163A (scavenger receptor cystein-rich type 1 protein M130) and MRC1 (mannose receptor 1) did not show an association with the loss of structural ECM proteins.

### Extracellular Protein Changes in Calcified and Symptomatic Plaques

**Figure 2.**
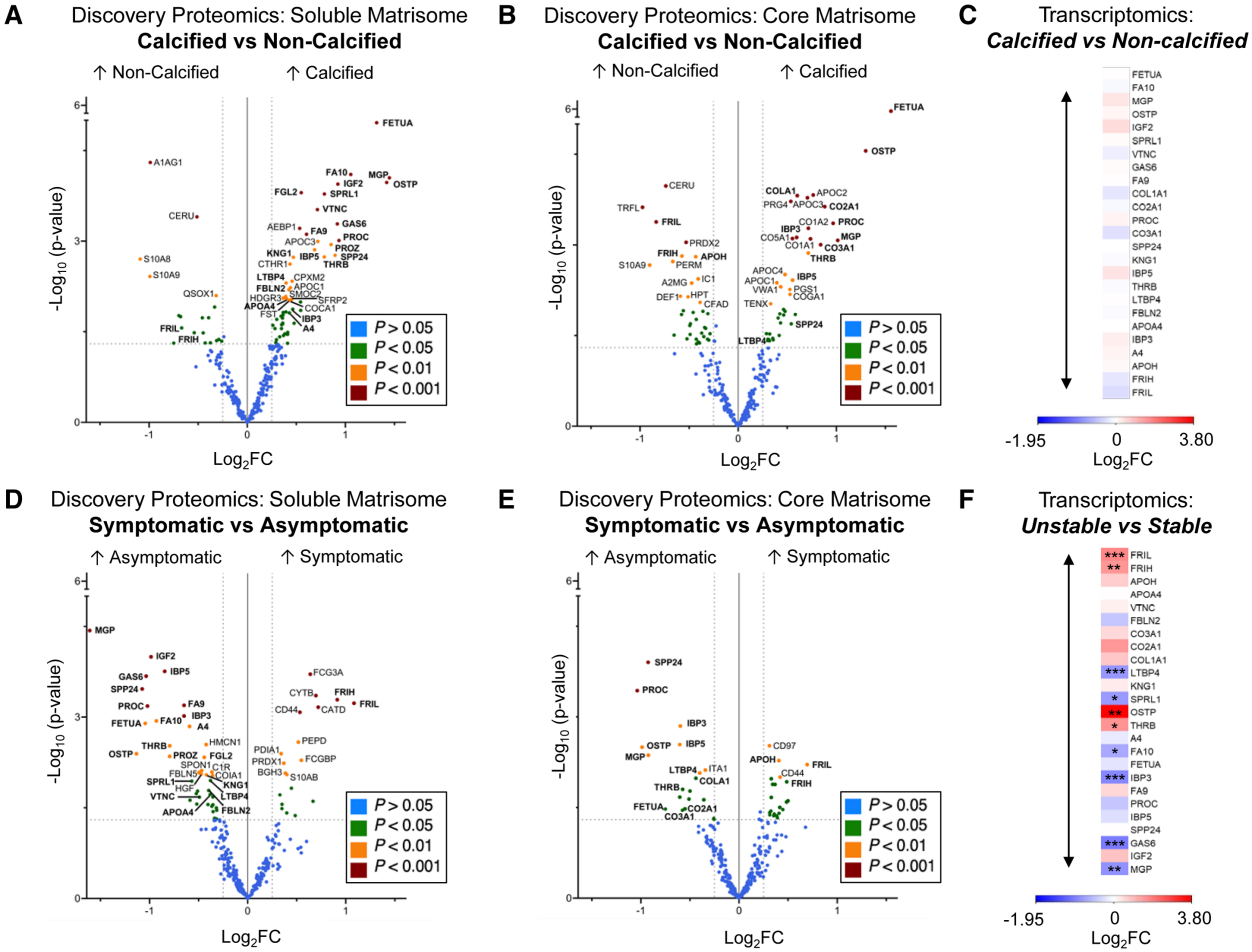
**Extracellular protein changes in calcified and symptomatic plaques. A** and B, Volcano plots of significantly dysregulated proteins in the calcified (n=60) vs noncalcified (n=46) core plaque comparison for soluble (NaCl; **A**) and core (GuHCl; **B**) matrisome extracts with 283 and 286 consistently quantified extracellular proteins, respectively. Proteins significant in both calcified vs noncalcified and symptomatic vs asymptomatic comparisons are labeled in bold. **C**, Heatmap displays the log2 fold changes (FC) between calcified (n=11) and fibroatheroma (n=9) plaques and nominal *P* values from the RNA-sequencing data set GSE104140 for the corresponding transcripts of proteins significantly changing in both calcified vs noncalcified and symptomatic vs asymptomatic comparisons. **D** and **E**, Volcano plots of significantly dysregulated proteins in the symptomatic (n=36) vs asymptomatic (n=69) comparisons of the plaque cores for the soluble matrisome (NaCl; **D**) and the core (GuHCl; **E**) matrisome extracts with 283 and 286 consistently quantified extracellular proteins, respectively. **F**, Heatmap displays the log2 FC between unstable (n=4) and stable (n=4) carotid plaques and nominal *P* values from the RNAseq experiment GSE120521 for the corresponding transcripts of proteins significantly changing in both symptomatic vs asymptomatic and calcified vs noncalcified comparisons. Differential protein analysis was conducted using the Ebayes method of the limma package correcting for age, sex, and statins. Differential expression analysis for RNAseq data was performed without corrections. Proteins with 0.01≤*P*<0.05, 0.001≤*P*<0.01, and *P*<0.001 are highlighted in green, orange, and red colors, respectively. Nominal *P* values are displayed in volcano plots. *P* values with multiple testing correction are provided in Summary Results and Statistics of the TMT Proteomics Using the NaCl Extract and Summary Results and Statistics of the TMT Proteomics Using the GuHCl Extract in the Supplemental Material. Log_2_FC indicates base 2 logarithm of fold change. Protein and gene names are denoted with Uniprot IDs.

To explore the impact of calcification on plaque proteome composition, we compared cores of calcified and noncalcified plaques for soluble and core matrisome proteins, respectively (Figure 2A and 2B). Among the most upregulated proteins in calcified plaques were FETUA (fetuin-A), OSTP (osteopontin), and gamma-carboxylated proteins such as MGP (matrix Gla protein), GAS6 (growth arrest-specific 6), FA9 and FA10 (coagulation factors IX and X), prothrombin (THRB), and vitamin-K–dependent proteins C and Z (PROC, PROZ). Additionally, an increase in C apolipoproteins (APOC1–4) was observed in calcified plaques. Conversely, inflammatory proteins, such as S10A8/A9 (calprotectin) and PERM (myeloperoxidase), and proteins associated with iron transport, including TRFL (lactotransferrin), FRIL and FRIH (ferritin light and heavy chain) and HPT (haptoglobin), were reduced. No corresponding changes were observed at transcript level in a publicly available bulk RNAseq data set comparing calcified (n=11) and noncalcified fibroatheromas (n=9; Figure 2C).

The proteomic signatures associated with calcification and inflammation were inversely correlated *(r*=−0.615 and *r*=−0.595 for soluble and core matrisome, respectively, both *P*<0.001). By conducting a comparative analysis between changes related to calcification (Figure 2A and 2B) and changes between symptomatic and asymptomatic patients (Figure 2D and 2E), we observed overlapping protein changes, which were labeled in bold font. Symptomatic plaques showed increased levels of FRIL and FRIH, CD44 (the hyaluronan receptor), and CD97 (a receptor essential for leukocyte migration).

After excluding calcified plaques (Figure S3), FRIL, FRIH, CATD (cathepsin D), and MMP9 (matrix metalloproteinase 9) remained the most upregulated proteins in symptomatic plaques. The upregulation of MMP9 and cathepsins is consistent with the findings of our previous proteomics study.^16^ The increase in FRIL and FRIH in symptomatic plaques was confirmed at the transcript level using a publicly available bulk RNAseq data set comprising stable and unstable carotid plaque samples (n=4 per group, Figure 2F). Additionally, gene expression of GAS6, MGP, IBP3 (insulin-like growth factor binding protein 3), and LTBP4 (latent-transforming growth factor beta-binding protein 4) was reduced in symptomatic plaques, consistent with the results of our present proteomics study.

Further correlation analysis was conducted between FRIL and FRIH with other serum proteins (Figure S4). In the plaque matrisome extracts, ferritin levels neither correlated with hemopexin and haptoglobin nor with albumin (*r*=−0.21 and *r*=−0.24, with *P*>0.05 for FRIH and FRIL, respectively). In the cellular SDS extracts, ferritin levels moderately correlated with hemoglobin (*r*=0.43 and *r*=0.39 for FRIH and FRIL, respectively). For a subset of the cohort (n=57), we also had measurements of serum ferritin. Ferritin levels in plaques only weakly correlated with ferritin levels in the circulation (*r*=0.28 and *r*=0.33 for FRIH and FRIL, respectively). This correlation was of similar strength as the correlation seen for hemoglobin and hematocrit. Thus, the increase in ferritin in plaques of symptomatic patients is not a result of increased contamination by serum proteins.

### Validation by Targeted Proteomics

To validate the findings from our untargeted proteomic analysis, we conducted targeted proteomics (parallel reaction monitoring [PRM]) to quantify 135 matrisome proteins by MS in the discovery cohort (Summary Details of Proteins and Peptides Quantified using Targeted Proteomics PRM Method and Summary Results and Statistics of the PRM Targeted Proteomics Analysis Using the GuHCl Extract in the Supplemental Material). Targeted proteomics results showed good agreement with those of the untargeted discovery approach, with correlations exceeding *r*=0.6 (*P*<0.001).

**Figure 3.**
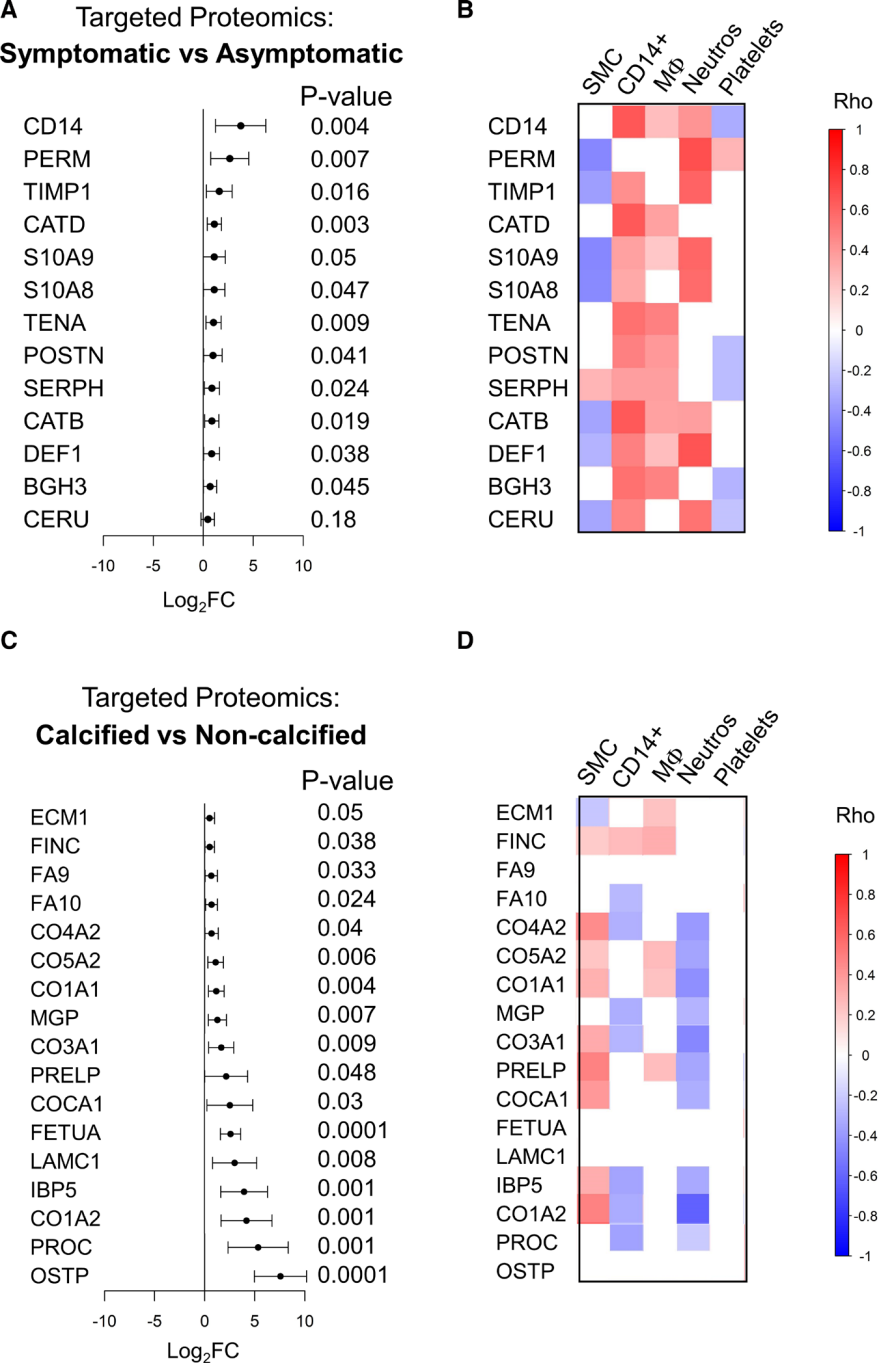
**Validation by targeted proteomics. A**, Forest plot depicting log2 fold changes (FC) and nominal *P* values of proteins validated by targeted proteomics in symptomatic (n=34) vs asymptomatic (n=64) core plaques. **B**, Spearman correlations of these proteins with the first principal component (PC) of cell clusters from our network analysis of intracellular proteins (Figure S5). **C**, Forest plot depicting log2 FC and nominal *P* values of proteins validated by targeted proteomics that were upregulated proteins in calcified (n=56) vs noncalcified (n=42) core plaques using targeted proteomics. **D**, Spearman correlations of these proteins with the first PC of cell clusters from our network analysis of intracellular proteins. Significant correlations are displayed with *P*<0.05 after Benjamini-Hochberg correction for multiple testing. CD14+ denotes CD14+ monocytes and cathepsin cluster. Differential protein analysis was conducted using the Ebayes method of the limma package correcting for age, sex, and statins. *P* values with multiple testing correction are provided in Summary Results and Statistics of the PRM Targeted Proteomics Analysis Using the GuHCl Extract in the Supplemental Material. BGH3 indicates transforming growth factor-beta-induced protein ig-h3; CATB, cathepsin B; CATD, cathepsin D; CD14, monocyte differentiation antigen CD14; CERU, ceruloplasmin; CO1A1, collagen alpha-1(I) chain; CO1A2, Collagen alpha-2(I) chain; CO3A1, Collagen alpha-1(III) chain; CO4A2, Collagen alpha-2(IV) chain; CO5A2, Collagen alpha-2(V) chain; COCA1, Collagen alpha-1(XII) chain; DEF1, neutrophil defensin 1; ECM1, extracellular matrix protein 1; FA9, coagulation factor IX; FA10, coagulation factor X; FETUA, fetuin-A; FINC, fibronectin; IBP5, insulin-like growth factor binding protein 5; LAMC1, Laminin subunit gamma-1; Log2FC, base 2 logarithm of fold change; MGP, matrix Gla protein; MΦ, monocytes/macrophages; Neutros, neutrophils; OSTP, osteopontin; PERM, myeloperoxidase; PRELP, prolargin; POSTN, periostin; SERPH, serpin H1; SMC, smooth muscle cell; TENA, tenascin; and TIMP1, metalloproteinase inhibitor 1.

Ranking changes in the inflammation signature in the plaque cores, based on their effect size revealed that CD14 and PERM showed the most pronounced changes (Figure 3A). Furthermore, PERM, TIMP1 (metalloproteinase inhibitor 1), S10A8/A9, and DEF1 (neutrophil defensin 1) were most strongly associated with neutrophils (Figure 3B), whereas the abundance of cathepsins (CATD, CATB) demonstrated a stronger correlation with macrophage markers (Figure 3B). This cellular distribution was further supported by network reconstruction analysis of the cellular proteome filtered for cell receptor-associated proteins, which revealed 9 significant clusters (Figure S5). Consistent with the M1 and M2 paradigm, the M1 cluster contains CD14, while the M2 cluster contains C163A and MRC1 (CD206). The strong association of cathepsins with CD14 suggests that cathepsins may be predominantly expressed in M1 macrophages. The neutrophil cluster contained several proteins that constituted the inflammatory signature of symptomatic plaques, including S10A8/A9, MMP9, and TIMP1.^11^

Next, we ranked the proteins of the calcification signature and identified OSTP and PROC as among the most upregulated proteins in cores of calcified plaques (Figure 3C). Notably, although most proteins of the calcification signature were positively correlated with SMCs and negatively with neutrophils, no significant correlations were observed between FA9, FETUA, and OSTP and any cellular marker proteins, indicating that these proteins were deposited rather than expressed in calcified plaques.

### Sex Differences in Plaques Attributed to Calcification

**Figure 4.**
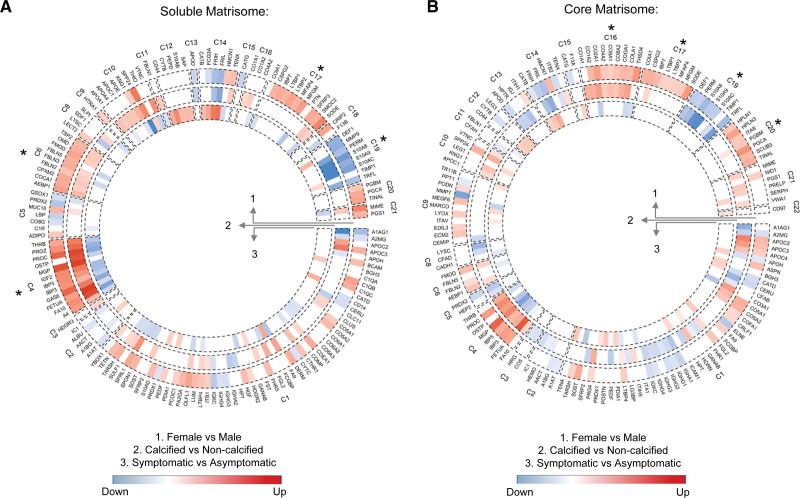
**Sex differences and their associations with calcification and inflammation.** Circular heatmap, showing the results of differential protein analysis for the soluble (NaCl; **A**) and core (GuHCl; **B**) matrisome extracts of plaque cores in 3 comparisons with 283 and 286 consistently quantified extracellular proteins, respectively (1) female (n=29) vs male (n=76) patients; (2) calcified (n=60) vs noncalcified (n=46) plaques and (3) symptomatic (n=69) vs asymptomatic (n=36) plaques. Differential protein analysis was conducted using the Ebayes method of the limma package correcting for age, sex, and statins (age and statins only for the sex comparison). The proteins are arranged in a circular graph based on the reconstructed matrisome and hierarchical clustering. Clusters enriched for dysregulated proteins in the sex comparison are marked with an asterisk (Fisher exact test, Benjamini-Hochberg–corrected for multiple testing *P*<0.05). *P* values with multiple testing corrections are provided in Summary Results and Statistics of the TMT Proteomics Using the NaCl Extract and Summary Results and Statistics of the TMT Proteomics Using the GuHCl Extract in the Supplemental Material. Protein names are denoted with Uniprot IDs.

Aside from calcification and symptomatic status, sex was identified as another important covariate in the proteomics analysis (Figure 1C). Given that previous studies have highlighted sex-specific gene expression changes in atherosclerosis,^20^ we compared sex-specific protein abundance changes in our study (Table S2). We reconstructed a matrisome network for the plaque cores and used hierarchical clustering to analyze the changes between females and males in all clusters (outer circle) for soluble proteins and the core matrisome, respectively (Figure 4A and 4B). Comparing sex differences to protein changes in calcified versus noncalcified plaques (middle circle), we found that the changes between plaques from female and male patients were highly correlated with the changes in calcification (*r*=0.808 and *r*=0.795 for log2 fold changes in soluble and core matrisome, respectively, both *P*<0.001). Conversely, sex differences were inversely associated with changes in symptomatic plaques (inner circle).

Four clusters of soluble matrisome proteins (Figure 4A: C4, C6, C17, C19) and 4 clusters of the core matrisome (Figure 4B: C16, C17, C19, C20) were significantly altered with sex. However, most of these protein changes were associated with calcification and inflammation, such as those in C4 containing gamma-carboxylated proteins or in C19 containing neutrophil-derived proteins. Only the sex-associated protein changes constituting C17 and C20 appeared to be independent of calcification and inflammation. C17 and C20 contained the 2 large-aggregating proteoglycans PGCA (aggrecan) and versican (CSPG2) and members of the HPLN (hyaluronan and proteoglycan link) protein family (HPLN1, HPLN3). As a result, the top significantly enriched pathways or GO (Gene Ontology) functional terms were growth-factor binding for C17 and cell adhesion and hyaluronic acid binding for C20 (Figure S6).

**Figure 5.**
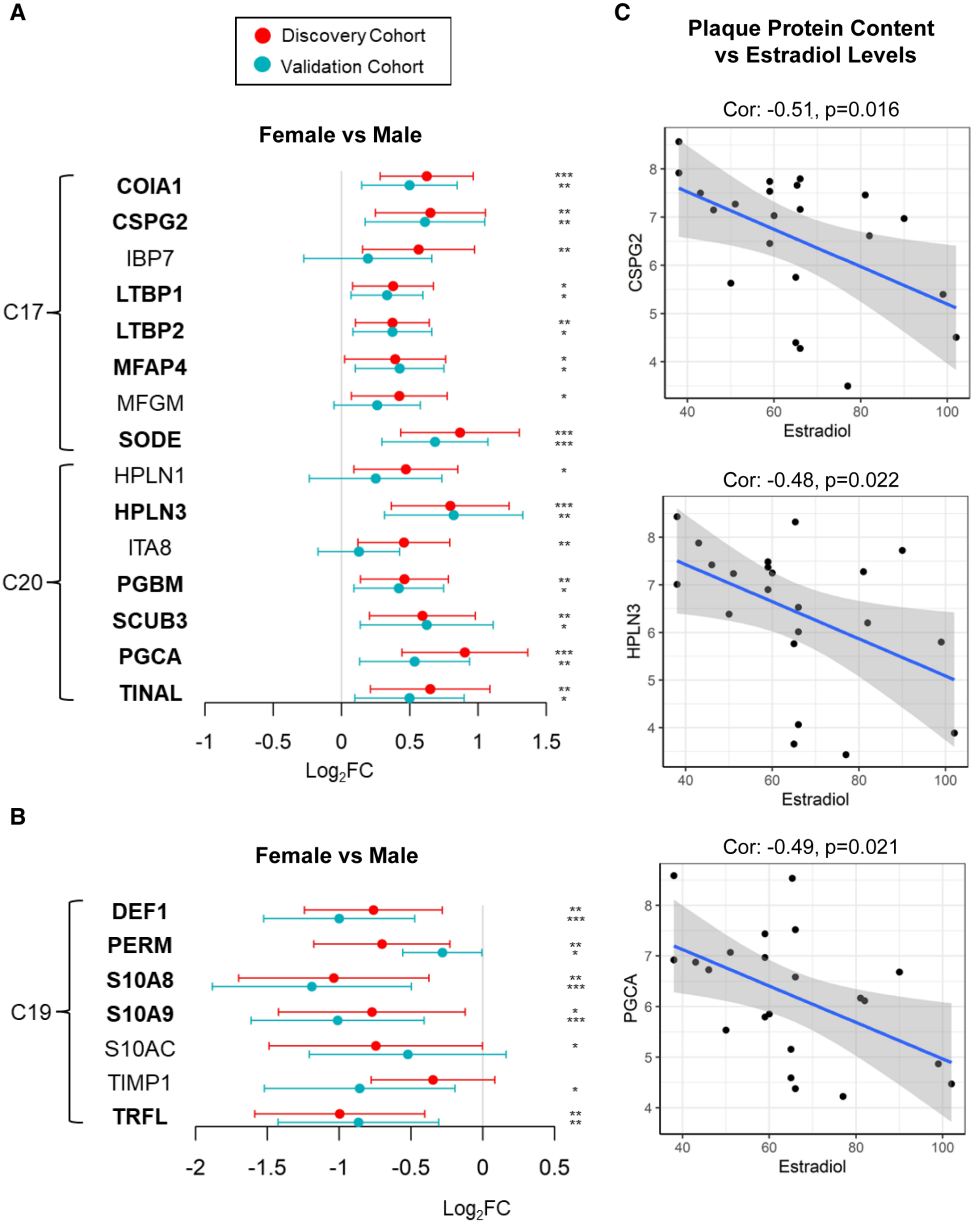
**Validation of sex-associated matrisome changes in the Athero-Express biobank.** Forest plot depicting log2 fold changes (FC), 95% CIs, and *P* values (Benjamini-Hochberg [BH] corrected for multiple testing) for the comparison between plaque cores of male and female patients in the core matrisome extract (GuHCl). The discovery cohort had 32 female vs 88 male patients. The validation cohort had 49 female vs 151 male patients. **A**, Proteins from the sex-associated matrisome clusters C17 and C20 (see Figure 4). **B**, Proteins from the inflammation signature from cluster C19 (see Figure 4). Proteins that were significantly changing between female and male patients in both cohorts appear in bold. Differential protein analysis for both cohorts was conducted using the Ebayes method of the limma package correcting for age and statins. *0.01≤*P*<0.05, **0.001≤*P*<0.01, and ***P<0.001. **C**, Association of 3 sex-associated matrisome proteins with serum estradiol measurements in the Athero-Express Biobank study. COIA1 indicates collagen alpha-1(XVIII) chain; CSPG2, versican; DEF1, neutrophil defensin 1; HPLN1, hyaluronan and proteoglycan link protein 1; HPLN3, hyaluronan and proteoglycan link protein 3; IBP5, insulin-like growth factor binding protein 5; IBP7, insulin-like growth factor-binding protein 7; ITA8, Integrin alpha-8; Log2FC, base 2 logarithm of fold change; LTBP1, latent-transforming growth factor beta-binding protein 1; LTBP2, latent-transforming growth factor beta-binding protein 2; MFAP4, microfibril-associated glycoprotein 4; MFGM, lactadherin; PERM, myeloperoxidase; PGBM, basement membrane-specific heparan sulfate proteoglycan core protein; PGCA, aggrecan; SCUB3, signal peptide, CUB and EGF-like domain-containing protein 3; SODE, extracellular superoxide dismutase; S10A8, protein S100-A8; S10A9, protein S100-A9; S10AC, protein S100-A12; TIMP1, metalloproteinase inhibitor 1; and TRFL, lactotransferrin.

We validated the sex-related changes in clusters 17 and 20 using label-free proteomics data from 200 carotid plaques of the Athero-Express Biobank (Table S3). Out of the 15 protein changes observed in relation to sex, 11 were confirmed, including SODE (superoxide dismutase), HPLN3, and the 2 large-aggregating proteoglycans CSPG2 and PGCA (Figure 5A). Moreover, both cohorts exhibited an inverse correlation between sex and inflammation (Figure 5B). Estradiol levels were available in a subset of female patients in the Athero-express Biobank (n=22), and analysis showed an inverse correlation with the CSPG2, HPLN3, and PGCA content of atherosclerotic plaques (Figure 5C), providing further evidence of the involvement of sex hormones in regulating plaque ECM composition.

### Integrating Proteomic Changes with Spatial and scRNAseq

**Figure 6.**
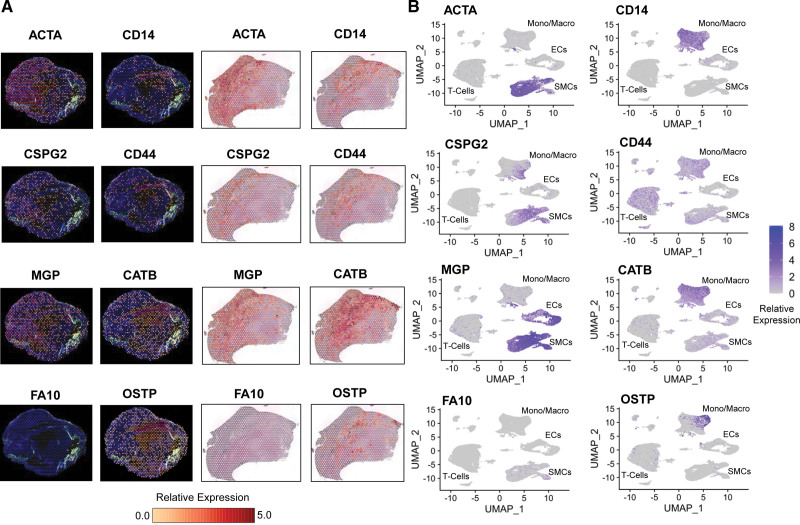
**Integration with spatial and single-cell RNA-sequencing (scRNAseq). A**, Spatial RNAseq: Feature heatmaps of expression levels using Loupe Browser. Selected genes included smooth muscle cell (SMC; *ACTA* [aortic smooth muscle actin]) and macrophage (*CD14* [monocyte differentiation antigen CD14]) markers and significantly changing extracellular proteins in discovery proteomics, including CSPG2 (versican), CD44 (hyaluronan receptor), MGP (matrix Gla protein), CATB (cathepsin B), FA10 (coagulation factor X), and OSTP (osteopontin). The expression levels of each cell from 2 plaques are shown using a yellow-to-red color scale. **B**, ScRNAseq: Unifold Manifold Approximation (UMAP) feature plots for selected cell markers (*ACTA, CD14*) and matrisome proteins are shown in the integrated scRNAseq data sets. Relative expression levels of each gene were calculated for spatial and scRNAseq data by dividing the read count of each cell/position by the total reads for that cell/position, multiplying with the Seurat scale factor, and performing natural logarithmic transformation. EC indicates endothelial cells.

Three publicly available scRNAseq data sets were integrated and visualized in Unifold Manifold Approximation plots before and after integration (Figure S7). The expression patterns of different regions in spatial RNAseq were visualized using feature plots (Figure S8). The combination of spatial RNAseq and integrated scRNAseq data analysis (Figure 6) revealed that only a subset of SMCs expressed high levels of CSPG2. Clustering analysis of scRNAseq data (Figure S9A) showed distinct clusters of SMCs, with *CSPG2* primarily expressed in SMC clusters 7 and 10 (Figure S9B), while SMC markers were primarily expressed by SMC cluster 3 (Figure S9E). Spatial RNAseq revealed that *CD14*^+^ cell–enriched regions were the main source of *OSTP* expression. scRNAseq detected *OSTP* expression in a subset of macrophages (cluster 5, Figure S9C). Despite being among the most significant changes detected by proteomics (Figure 3), neutrophil transcripts (*PERM, DEF1*), *FA9*, and *FETUA* were undetectable by scRNAseq (Figure S9C and S9D).

Further analyses were performed to characterize the macrophage clusters by performing one versus all clusters analysis and reporting the top-5 upregulated transcripts for each cluster (Figure S10B). Cluster 4, 5, and 8 were the main clusters of macrophages. Cluster 4 were M2 macrophages (*C163A* and *MRC1* high; Figure S9E) that differed from other macrophage clusters by increased expression of *SELENOP* (selenoprotein P), macrophage inflammatory protein 1 beta (*CCL4L2*) and several complement factors. High *OSTP* (*SPP1* gene) expression characterized the macrophages in cluster 5. The CellChat tool was used to further study cell-cell communication patterns. *OSTP* was predominantly implicated in the outgoing communication of Cluster 5 macrophages via *CD44* (Figure S10C and S10D). The hyaluronan receptor CD44 was increased in symptomatic lesions (Figure 2D). The macrophages in cluster 8 were characterized by an upregulation of calprotectin (S10A8/A9).

### Comparison to Imaging Classification by Ultrasound

**Figure 7.**
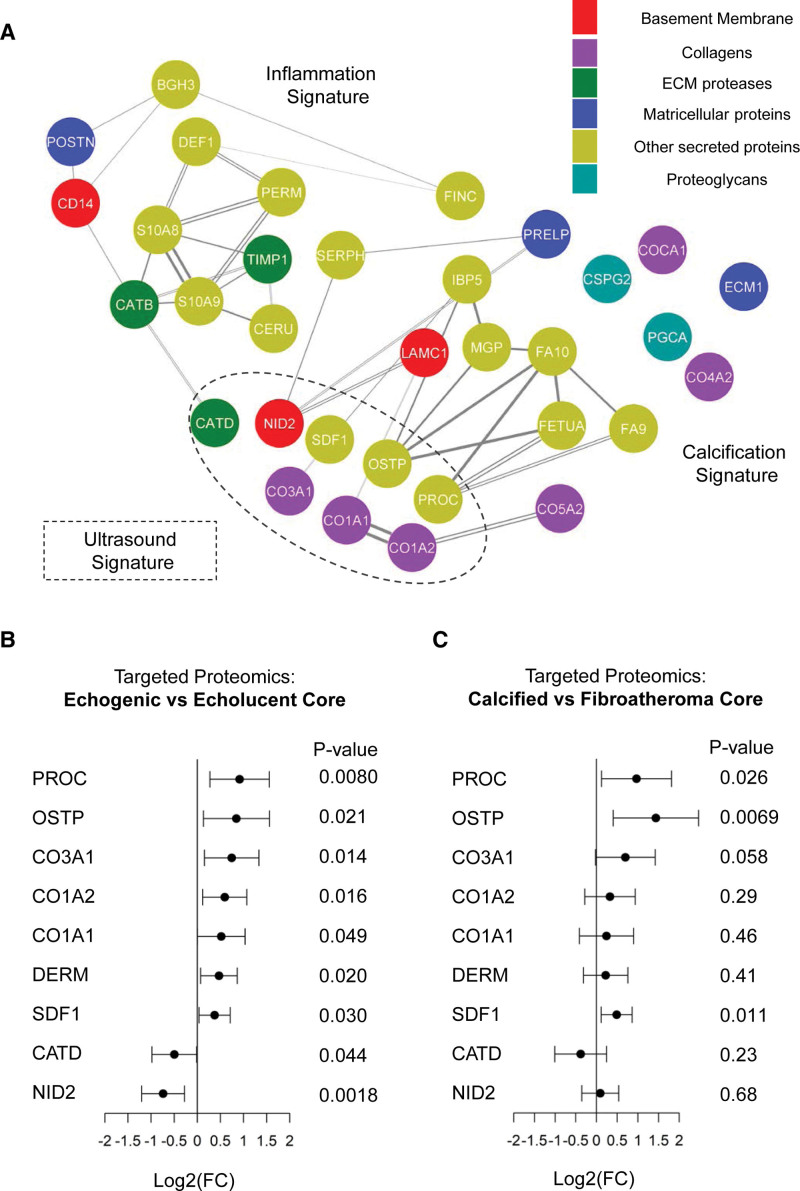
**Ultrasound classification vs calcification and inflammation signatures. A**, Part of the reconstructed matrisome network from the discovery cohort. Nodes include validated significant changes of extracellular proteins in symptomatic vs asymptomatic, calcified vs noncalcified, or echogenic vs echolucent comparisons. Nodes are colored based on the functional category of each protein. The line thickness is proportional to the conditional mutual information between the 2 proteins. Double lines represent experimentally verified protein-protein interactions. **B**, Forest plot depicting log2 fold changes (FC) with significant nominal *P* values in targeted proteomics (135 extracellular proteins in total) in the comparison of echogenic (n=43) vs echolucent (n=28) plaque cores. **C**, Forest plot depicting the log2 FC with nominal *P* values targeted proteomics (135 extracellular proteins in total) in the comparison of calcified (n=10) vs fibroatheroma (n=19) plaque cores based on the histological characterization of the validated ultrasound signature. Differential expression analysis was conducted using the Ebayes method of the limma package correcting for age, sex, and statins. CATD indicates cathepsin D; DERM, dermatopontin; ECM1, extracellular matrix protein 1; FETUA, fetuin-A; IBP5, insulin-like growth factor binding protein 5; Log^2^(FC), base 2 logarithm of fold change; OSTP, osteopontin; POSTN, periostin; SDF, stromal cell-derived factor; and SERPH, serpin H1.

The reconstructed matrisome correlation network in Figure 7A depicts the plaque proteins associated with the inflammation and calcification signatures and their relationship to the current ultrasound classification. Higher levels of CATD and NID2 (nidogen 2), a basal lamina protein, were observed in echolucent plaques, whereas echogenic plaques were characterized by increased levels of calcification-related proteins (PROC, OSTP), fibrillar collagens (CO1A1 [collagen alpha-1(I) chain], CO1A2 [collagen alpha-2(I) chain]), DERM (dermatopontin), and SDF1 (stromal cell-derived factor 1; Figure 7B). Comparing calcified lesions versus fibroatheroma based on histology (Figure 7C) confirmed an increase in PROC, OSTP, and SDF1 levels. Thus, echogenic plaques reflect predominantly calcified lesions.

### Identifying Molecular Plaque Phenotypes Based on Proteomics

**Figure 8.**
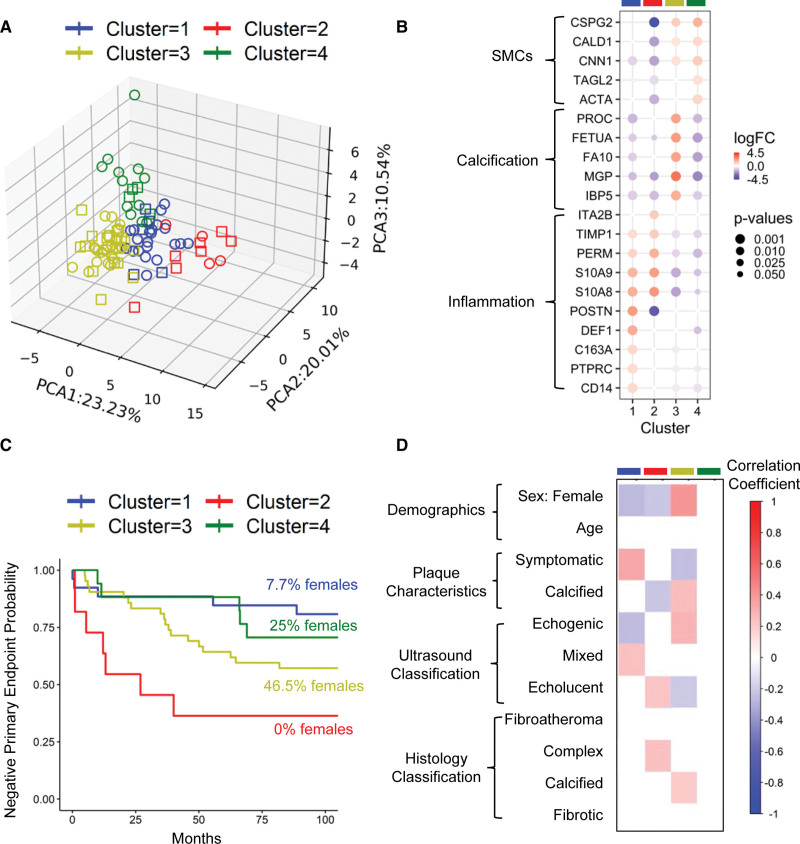
**Molecular plaque phenotypes and cardiovascular outcomes. A**, Representation of the plaque cores using principal component analysis (PCA) and their clustering using KMEANS. Rectangles represent cases with a primary cardiovascular end point over a 9-year follow-up. **B**, Top-5 proteins upregulated in each cluster using one-versus-all clusters differential expression analysis with the Ebayes method of the limma package (*P* values corrected for multiple testing). **C**, Kaplan-Meier plot for the survival analysis of patients with the 4 distinct molecular plaque phenotypes based on the primary composite cardiovascular end point. **D**, Correlation of the proteomics clusters against demographics, imaging, and histological characteristics. Point-biserial correlation was used for binary features and Spearman correlation for continuous ones. Significant correlations are displayed with *P*<0.05 after BH correction for multiple testing. ACTA indicates aortic smooth muscle actin; CALD1, caldesmon; CD14, monocyte differentiation antigen CD14; CNN1, calponin-1; CSPG2, versican; C163A, scavenger receptor cysteine-rich type 1 protein M130 ; DEF1, neutrophil defensin 1; DERM, dermatopontin; FA10, coagulation factor X; FETUA, fetuin-A; IBP5, insulin-like growth factor binding protein 5; ITA2B, integrin alpha IIB; logFC, base 2 logarithm of fold change; MGP, matrix Gla protein; PERM, myeloperoxidase; POSTN, periostin; PTPRC, receptor-type tyrosine-protein phosphatase C; PROC, vitamin K-dependent protein C; SMC, smooth muscle cells; S10A8, protein S100-A8; S10A9, protein S100-A9; TAGLN, transgelin; and TIMP1, metalloproteinase inhibitor 1.

Through the integration of proteomic measurements of both the cellular and matrisome proteome, principal component analysis and clustering analyses were performed to identify molecular plaque phenotypes. The analysis resulted in 4 distinct clusters (Figure 8A), with principal component 1 showing an inverse association with structural ECM proteins and principal component 2 showing a negative association with the calcification signature. Furthermore, principal component 3 exhibited a positive association with SMC markers but a negative association with CD14 and OSTP. To further characterize the clusters, we performed one versus all comparisons of their protein abundances, and the top-5 upregulated proteins for each cluster are presented in Figure 8B. Clusters 1 and 2 included predominantly plaques from male patients with a high degree of inflammation. Yet, cluster 2 had markedly worse outcomes (Figure 8C). Cluster 2 was separated from cluster 1 by a loss of SMC markers, reduced abundance of CSPG2, and an increase in the platelet-specific ITA2B (integrin alpha IIB). Clusters 3 and 4 included most female patients, had less inflammation, but a higher SMC and CSPG2 content. Cluster 3, however, differed by the higher abundance of proteins linked to calcification.

Although classification based on ultrasound and histology did not achieve an area under the curve (AUC) >50.0% in the discovery cohort, unsupervised clustering based on proteomics achieved an AUC of 60.4% (Table S4). Cluster 2 had the strongest association with adverse cardiovascular outcomes (Figure 8C) and consisted only of males with echolucent plaques and reduced structural ECM proteins (Figure 8B and 8D). However, most females were grouped into cluster 3, which was also strongly associated with adverse cardiovascular events despite representing calcified, echogenic plaques with high CSPG2 content. Cluster 1, representing symptomatic mixed plaques, and cluster 4, which represented SMC-rich plaques, were associated with a better prognosis.

To explore whether plasma measurements could identify plaque clusters, we expanded our previously identified inflammation signature^11^ (MMP9, CATD, FN1 [fibronectin], calprotectin, or S10A8/9) with PERM, which was one of the most upregulated proteins in symptomatic plaques in our targeted proteomics analysis. We trained and tested elastic-net logistic regression models to predict the plaque proteomic clusters by combining the inflammatory plasma biomarkers, with the ultrasound classification of plaques and the available clinical information from the patients. Among the clinical, ultrasound, and plasma biomarkers, all 5 inflammatory plasma biomarkers were selected using the elastic-net approach. Results of 10-fold cross-validation revealed that the logistic regression models were able to predict plaque clusters with 82.29% AUC.

Finally, we utilized a multiobjective evolutionary optimization algorithm to identify the minimum plaque signature that performs best as input for a nonlinear classifier (support vector machines). A 4 protein signature, consisting of CNN1, PROC, the collagen-binding protein SERPH1 (serpin H1), and CSPG2, returned an AUC of 75.0% using 10-fold cross-validation. When tested on the Athero-Express Biobank study, the proteomics signature outperformed the histology-based Plaque Vulnerability Index with an AUC of 67.5% compared with 51.0% (Table S4).

## DISCUSSION

This study represents the most comprehensive proteomics analysis of human atherosclerotic plaques to date, incorporating proteomics analyses of 419 fresh-frozen plaque samples from 320 patients, integrated with bulk, scRNAseq, and spatial RNAseq, carotid ultrasound, and histology. The analysis linked inflammation and calcification signatures to clinical characteristics, such as sex, and validated prognostic signatures for cardiovascular outcomes in an independent cohort. Unlike previous studies that relied on bulk RNAseq data,^8^ our study is the first to use the plaque proteome to define plaque clusters and annotate them based on clinical characteristics and cardiovascular outcomes.

### Proteomics of Human Atherosclerosis

Prior proteomics studies on carotid plaques^11,14–19^ were limited to less than 20 patients and conducted label-free proteomics analysis. Matic et al^19^ were among the first to apply TMT multiplexing to carotid plaques from 18 patients. Larger studies are needed to identify plaque subtypes. The largest previous proteomic study^21^ on coronary plaques used autopsy samples. The use of fresh, frozen samples and the focus on ECM in the present study doubled the coverage of the human plaque proteome. Core and periphery samples were separated as the 2 regions differed in their proteomic profile and periphery samples can have a variable contribution from the normal vessel wall.

### Inflammation Signatures of Atherosclerosis

Inflammatory proteins play an important role in atherogenesis.^22^ In line with our previous results, the inflammatory signature of symptomatic plaques included MMP9 and cathepsins.^11^ MMP9 was correlated with neutrophils, whereas cathepsins were associated with macrophages. The importance of neutrophils in advanced atherosclerosis is further highlighted by PERM as being one of the most significantly upregulated proteins in our targeted proteomics analysis of symptomatic plaques. As shown by Silvestre-Roig et al,^22^ neutrophils determine plaque stability and induce SMC apoptosis on contact. Neutrophil transcripts are particularly prone to degradation. Thus, neutrophil RNA and protein changes were not concordant. Another striking observation was the rise in ferritin along with other proteins involved in iron transport. Ferritin can promote plaque destabilization through the oxidation of lipids by iron overload.^23^ Elevated body iron stores have been previously associated with increased risk of carotid atherosclerosis.^23^ Ferritin changes in plaques, however, were not due to serum contamination. Both scRNAseq and spatial RNAseq data confirmed that ferritin is expressed by plaque macrophages. Although RNA reflects gene transcription at the time of tissue collection, extracellular proteins tend to accumulate over time, and protein deposition is better captured by proteomics.

### Calcification Signatures of Atherosclerosis

Consistent with the notion that calcification dampens vascular inflammation, the proteomics signatures linked to plaque calcification and inflammation were inversely correlated. Vascular calcification results from an imbalance between stimulatory mediators and calcification inhibitors, such as MGP^24^ and FETUA.^25^ The upregulation of both proteins in calcified plaques suggests a compensatory increase of calcification inhibitors. In addition to MGP, proteomics identified the upregulation of other gamma-carboxylated proteins in calcified plaques, such as FA9 and FA10. Gamma-carboxylation is a key calcification regulator at cellular level.^26^ This vitamin K–dependent post-translational modification replaces glutamate residues with gamma-carboxyglutamate. Since no patient was under warfarin treatment, an oral vitamin K antagonist, the observed differences in gamma-carboxylated proteins are not related to medication. Several gamma-carboxyglutamate residues are contained within the Gla domain of certain proteins, responsible for the high-affinity binding of calcium ions. Thus, Gla domain-containing proteins may be retained in calcified plaques. This is consistent with the observation that transcriptomics data revealed no corresponding changes in gene expression for FA10 and FETUA.

Typically, there is a poor yield of RNA from calcified lesions. Karlöf et al^27^ performed a high versus low calcification comparison on carotid plaques using microarray data from 40 patients. When comparing their top significant transcriptomic changes to our proteomic changes, abundant collagens (CO1A1 and CO1A2) and PRG4 (proteoglycan 4) were found to be significantly upregulated both at the transcript and the protein level. In contrast, APOC1 was increased in the proteome but reduced in the transcriptome of calcified plaques, consistent with a potential plasma origin. When exploring changes between symptomatic and asymptomatic plaques using only noncalcified plaques, PROC and FETUA were upregulated in asymptomatic plaques, suggesting a role in the early calcification response or plaque stability.

### Sex Differences in Atherosclerosis

Although the pathobiology of atherosclerotic cardiovascular disease is generally similar between both sexes, there are unique aspects specific to women, with the coronary artery calcium scoring, for instance, predicting mortality better in women than in men.^28^ In a recent study^20^ using RNAseq, sex-stratified gene regulatory network analysis identified GAS6 and serpin G1 as potential key SMC driver genes in plaques from females compared with males. In our cohort, GAS6 was also more abundant in plaques of female patients, but serpin G1 was unchanged at the protein level. GAS6, however, was part of the calcification signature, and calcification was more common in carotid plaques from female patients. When further comparisons were restricted to noncalcified plaques, GAS6, and other calcification-related proteins were not different between plaques from male and female patients. Thus, these changes with sex might be explained by a higher susceptibility to calcification in age-matched female patients. When excluding protein changes associated with calcification, only clusters containing the 2 large-aggregating proteoglycans, CSPG2 and PGCA, were found to be sex-related. When calcified plaques were excluded, differences in PGCA and CSPG2 remained as the protein changes most prominently associated with sex along with hyaluronic acid–binding link proteins HPLN1 and HPLN3 (data not shown). These sex-related matrisome changes were validated in the Athero-Express cohort.^12^

### The Role of Large, Aggregating Proteoglycans in Atherosclerosis

CSPG2 and PGCA are expressed in a subset of SMCs in human carotid plaques. Large, aggregating proteoglycans play an important role in regulating the mechanical properties of the vessel wall^29^ by controlling tissue hydration and vascular wall viscosity. CSPG2 is known to contribute to lipoprotein retention in atherosclerosis. In murine atherosclerosis, Hartmann et al^30^ identified a transition state SMC phenotype linked with plaque stability that expressed ECM proteins and PGCA. We have recently uncovered an increase in CSPG2 and PGCA in stented porcine coronary arteries.^31^ Proteases of the ADAMTS (a disintegrin and metalloproteinase with thrombospondin motifs) family partly control the accumulation of large proteoglycans after vascular injury.^29^ Proteoglycan deposition could represent a missing link between lipoprotein retention and hemodynamics, consistent with the known predilection sites for atherosclerosis. In this study, we report differences in hyaluronan-binding proteoglycans in carotid plaques between males and females. This observation was further supported by an inverse association of the hyaluronan-binding proteoglycan content in plaques to circulating estradiol levels in female patients.

### Plaque Classification by Ultrasound

Carotid duplex ultrasound is commonly used as a first-line diagnostic tool to assess extracranial carotid artery stenosis.^32^ Echolucent plaques are associated with an increased risk for future ischemic cerebrovascular events.^33^ Carotid plaque area as well as plaque neovascularization detected in carotid ultrasound are also predictive for future cardiovascular events.^34^ However, this imaging prediction is mainly based on the separation of advanced from early stages of atherosclerosis. In our study, all patients had advanced atherosclerosis. The proteome changes corresponding to echogenic plaques were predominantly fibrillar collagens and few proteins of the calcification signature. Although ultrasound imaging captures the collagen and lipid content of the plaque, it is not able to distinguish between complex lesions with high inflammation and stable fibroatheroma with lipid deposits and low-grade inflammation. Thus, proteomic signatures could complement carotid imaging read-outs to improve prognostication.

### Plaque Classification by Histology

Histological evaluation with American Heart Association classification is performed after surgery to assess morphological properties of atherosclerotic plaques.^35^ Although there are some data regarding histological plaque properties and patient outcome in contemporary cohorts, such as the ability of a histology-based Plaque Vulnerability Index to predict outcomes,^36^ symptomatic carotid stenosis is defined as the occurrence of symptoms ipsilateral to stenosis during the preceding 6 months.^32^ Given that atherosclerotic plaque morphology is a dynamic process, most plaques undergo several subclinical ruptures and healings at the stage of advanced lesions.^37^ Therefore, the in-depth molecular characterization offered by proteomics may better capture the evolution of plaques over time. Expanding on the traditional classification of vulnerable and nonvulnerable plaques, male patients with molecular features of vulnerable plaques (cluster 2) had the worst prognosis followed by a subgroup of predominantly female patients with calcified lesions (cluster 3) with large-aggregating proteoglycans driving most of the clustering. Histology captured only a subset of these proteomic changes. Thus, assessment of plaque proteome content even at a single location, together with clinical risk stratification may improve identification of vulnerable patients at risk of future cardiovascular events.

### Risk of Recurrent Events

Atherosclerosis is a systemic disease. Patients with atherosclerosis at multiple vascular beds are at particularly high risk for atherothrombotic events, and each affected territory increases the risk not only of local organ damage but also the total risk of cardiovascular events.^32^ This warrants more aggressive treatment of cardiovascular risk factors for secondary prevention in these patients.^32^ In a previous study, plaque calcification and plaque eccentricity were positively correlated to plaque burden in both peripheral and coronary artery disease.^38^ We show here that proteomic characteristics of carotid plaques are associated with long-term cardiovascular events. Echolucent plaques with reduced structural ECM proteins had the worst prognosis (cluster 2), followed by calcified plaques (cluster 3). Although calcified plaques are traditionally considered stable lesions with low risk of embolization, the incidence of preoperative neurological symptoms was similar in patients with calcified and noncalcified plaques.^39^ Similarly, recent findings suggest that stable calcified plaques in coronary arteries are associated with high cardiovascular risk.^40^ In our cohort, calcified plaques were more common in female patients, highlighting potential sex differences also in advanced stages of atherosclerosis. A signature of 4 proteins, as revealed by machine learning predicted the composite cardiovascular end point with high accuracy in both the discovery and validation cohort over a 9 and 3-year follow-up periods, respectively. The signature included the SMC marker CNN1, the gamma-carboxylated protein PROC, the collagen-binding protein SERPH1 and the hyaluronan-binding proteoglycan CSPG2. Although plaque ECM proteins may not be readily detectable in the circulation, inflammatory plasma proteins may aid in classifying patients. The identified plaque clusters could be predicted using inflammatory plasma biomarkers (MMP9, CATD, FN1, calprotectin or S10A8/9, PERM) in combination with clinical characteristics (82.29% AUC, using 10-fold cross-validation).

### Study Limitations

Carotid endarterectomies represent advanced atherosclerotic plaques, which differ from early atherosclerosis. Moreover, all patients in the study were white patients. In addition, future comparisons should include plaques from different locations within the arterial bed, which have been associated with distinct genetic susceptibility loci.^41^ Coronary plaques, however, are more difficult to attain than fresh-frozen samples. Furthermore, MS has limitations in detecting very low-abundant proteins. Finally, our spatial RNAseq analysis was limited by the small sample size (n=2).

### Conclusions

In the largest proteomics data set of fresh-frozen human plaques to date, we combined untargeted and targeted proteomics with network reconstruction and clustering techniques to provide molecular insights into protein changes related to clinical characteristics. Our findings demonstrate the potential of proteomics to complement histology and imaging modalities for improved phenotyping of stable and unstable lesions. Furthermore, our results suggest that hyaluronan-binding proteoglycans exhibit a sex-specific dysregulation pattern, are inversely associated with serum estradiol, and contribute to risk stratification of patients into clinically relevant groups that are associated with cardiovascular outcomes. Future studies should investigate whether the accumulation of hyaluronan-binding proteoglycans contributes to the accelerated progression of atherosclerosis in postmenopausal women.

## ARTICLE INFORMATION

### Acknowledgments

Spatial transcriptomics was performed at the Core Facilities of the Medical University of Vienna, a member of Vienna Life-Science Instruments (VLSI). We would also like to acknowledge the contribution of Dr Marion Groeger from the Core Facilities of the Medical University of Vienna in Austria. The authors also like to thank all the (former) employees involved in the Athero-Express Biobank Study of the Departments of Surgery of the St. Antonius Hospital Nieuwegein and University Medical Center Utrecht for their continuing work. Lastly, we would like to thank all participants of the Athero-Express Biobank Study; without them, these kinds of studies would not be possible. Additional Information: Coauthor Prof. Marion Groeger, died April 14, 2022.

### Sources of Funding

M. Mayr is a British Heart Foundation (BHF) Chair Holder (CH/16/3/32406) with BHF program grant support (RG/F/21/110053). K. Theofilatos and M. Mayr were also supported with a BHF project grant (PG/20/10387). M. Mayr received support from the Leducq Foundation (“PlaqOmics,” 18CVD02). M. Mayr is also supported by the Vascular Ageing and Clinical Stroke Research center (VASCage). VASCage is a Competence Center for Excellent Technologies (COMET) within the COMET program and funded by the Federal Ministry for Climate Action, Environment, Energy, Mobility, Innovation, and Technology, the Federal Ministry of Labor and Economy, and the federal states of Tyrol, Salzburg, and Vienna. COMET is managed by the Austrian Research Promotion Agency (Project number: 898252). J. Barallobre-Barreiro was supported by a BHF Intermediate Fellowship (FS/19/33/34328). This work was supported by the Anniversary Fund of Austrian National Bank grant to Johann Wojta, Austrian National Bank (OeNB) grant number: AP15688ONB. Furthermore, the work was supported by the Association for the Promotion of Research in Arteriosclerosis, Thrombosis, and Vascular Biology. S.W. van der Laan is funded through European Union Horizon 2020 (EU H2020) TO_AITION (grant number: 848146). We are thankful for the support of the Netherlands CardioVascular Research Initiative of the Netherlands Heart Foundation (CVON 2011/B019 and CVON 2017-20: Generating the best evidence-based pharmaceutical targets for atherosclerosis [GENIUS I&II]), the European Research Area Network on Cardiovascular Diseases (ERA-CVD) program “druggable-MI-targets” (grant number: 01KL1802), and the Leducq Fondation “PlaqOmics.”

### Disclosures

S.W. van der Laan has received Roche funding for unrelated work. The other authors report no conflicts.

### Supplemental Material

Supplemental Methods

Summary Proteomics and Statistics Results of TMT Proteomics Data on SDS Extract.

Summary Results and Statistics of the TMT Proteomics Using the NaCl Extract.

Summary Results and Statistics of the TMT Proteomics Using the GuHCl Extract.

Differential Expression Results Comparing Clusters of Spatial RNA-Sequencing Data From Carotid Plaques

Summary details of Proteins and Peptides Quantified Using Targeted Proteomics PRM Method

Summary Results and Statistics of the PRM Targeted Proteomics Analysis Using the GuHCl Extract

Major Resources Table

Tables S1–S4

Figures S1–S10

References 42–51

## Supplementary Material


