# Supplementary material for: Proteomic Atlas of Atherosclerosis: The Contribution of Proteoglycans to Sex Differences, Plaque Phenotypes, and Outcomes
Source: Circ Res. 2023 Aug 30;133(7):542–58. doi: 10.1161/CIRCRESAHA.123.322590 (PMC10498884; doi:10.1161/CIRCRESAHA.123.322590)
Supplement: Supplementary file 2 [file res-133-542-s002.docx]

**SUPPLEMENTAL MATERIAL**

**A Proteomic Atlas of Atherosclerosis:**

**The Contribution of Proteoglycans to Sex Differences, Plaque Phenotypes and Outcomes**

[**Supplemental Methods** 1](#_Toc139602985)

[Patient population. 1](#_Toc139602986)

[Carotid plaque morphology. 2](#_Toc139602987)

[Protein extraction. 3](#_Toc139602988)

[Deglycosylation. 4](#_Toc139602989)

[In-solution digestion. 4](#_Toc139602990)

[Multiplexed proteomics workflow in the discovery cohort. 5](#_Toc139602991)

[Targeted proteomics workflow. 6](#_Toc139602992)

[Label-free proteomics in the Athero-Express Biobank. 7](#_Toc139602993)

[Statistical and bioinformatics analysis. 9](#_Toc139602994)

[Major Resources Table 11](#_Toc139602995)

[Data & Code Availability 11](#_Toc139602996)

[Online Supplemental Files 12](#_Toc139602997)

[Supplemental Tables 12](#_Toc139602998)

[Table S1. Clinical characteristics of the patient cohort. 13](#_Toc139602999)

[Table S2. Clinical characteristics of the discovery cohort in the sex comparison. 15](#_Toc139603000)

[Table S3. Clinical characteristics of the validation cohort (Athero-Express) in the sex comparison. 17](#_Toc139603001)

[Table S4. Machine learning analysis for the prediction of the follow-up primary endpoint. 18](#_Toc139603002)

Supplemental Methods

Patient cohorts.

120 consecutive patients with carotid artery stenosis undergoing carotid endarterectomy were included in the study as a discovery cohort. All surgical procedures were performed at the Department of Vascular Surgery, Medical University of Vienna, Austria. The indication for surgery included symptomatic carotid artery stenosis or high-grade asymptomatic stenosis (> 70%). The degree of luminal narrowing was determined by carotid Duplex ultrasound and/or computed tomography angiography using the criteria of the North American Symptomatic Carotid Endarterectomy Trial.^42^ Patients were considered symptomatic if they had experienced a stroke, transient ischemic attack, or amaurosis fugax ipsilateral to the carotid artery stenosis within 6 months before carotid endarterectomy. Patients with hemorrhagic, lacunar, or cardioembolic stroke were excluded from the study. Lacunar strokes were defined as the presence of characteristic primary motor, primary sensory, or sensory-motor symptoms in combination with deep white-matter lesions or basal ganglia lesions 1 cm or less in diameter. Suspected cardioembolic stroke, in patients with atrial fibrillation, recent myocardial infarction, unstable angina, recent congestive heart failure, and valvular disease prone to production of emboli, was reviewed by an independent, experienced cardiologist.

9-year follow-up was collected for all patients and the primary cardiovascular endpoint was defined as the composite of cardiovascular death, myocardial infarction, transient ischemic attacks or stroke as well as atherosclerosis progression in the coronary or peripheral arteries requiring either interventional (percutaneous coronary intervention or peripheral balloon angioplasty with and without stenting) or surgical revascularization (aortocoronary bypass or peripheral bypass). 41 patients reached primary endpoint in the 9-year follow-up. The study has been reviewed and approved by the Ethics Committee of the Medical University of Vienna, and all study subjects gave written informed consent.

Following carotid endarterectomy, carotid atherosclerotic plaques were excised and visually inspected. Plaques were divided transversely at the site of maximum stenosis, one half was sent for histologic analysis and the other was used for proteomics analysis. The site of maximal stenosis was identified as the plaque core, which was confirmed by histologic analysis of adjacent sections. The site of the plaque most distant to the plaque core, was defined as plaque periphery (Extended Data *Fig.* *1A*). Following dissection, plaque core and periphery samples were immediately snap-frozen in liquid nitrogen and stored at -80˚C for further analysis. A total of 219 carotid endarterectomy samples (110 core and 109 periphery samples) were analysed.

Carotid plaque morphology.

The morphological evaluation of carotid artery stenosis before surgery was assessed using carotid Duplex ultrasound and/or computed tomography angiography^.6^ Plaques were classified according to echogenicity into echolucent (non-calcified), echogenic (calcified), and mixed plaques (partly calcified) in the expert vascular laboratory at the Medical University of Vienna. For further validation of the ultrasound characterization of the plaques, computed tomography angiography was applied to classify plaques into non-calcified, calcified, and partly calcified (mixed) plaques and an agreement of 86% was achieved between Duplex ultrasound and computed tomography angiography characterization of the plaques.

For histological classification endarterectomy specimens were formalin-fixed and embedded in paraffin. Transverse sections were cut each 3mm along the plaque by experienced technicians. Specimens were stained with hematoxylin-eosin and elastic-van-Giessen staining and classified according to modified AHA classification based on morphological description.^43^ Finally, the plaques were characterized as calcified and non-calcified based on the assessments of the surgeon and the pathologist. Computed tomography angiographies (CTA) from 29 patients were used to verify the calcification classification of the plaques. In order to quantify plaque density, we used a modified Agatston score with an attenuation threshold of 600 Hounsfield Units (HU). The threshold was determined via Region Of Interest (ROI) measurements and ensured the registration of complete visually visible plaque area and prevented the inclusion of iodine. Due to the retrospective calcium scoring and consequently, heterogeneity of CT protocols, we categorized exams and referred patients to either protocol A or B. Protocols used different scanning and reconstruction parameters, thus altering the resulting score. Four patients needed to be excluded from the analysis, two of them not meeting the protocol criteria, two of them because iodine enhancement exceeded the predetermined HU-threshold. Plaques within a range of 2 cm above and below the carotid bifurcation were included for scoring. For analysis we used a commercially available software (SyngoVia CaScoring, Siemens Healthineers, Forchheim, Germany). The independent binary classification of the plaque calcification status by a surgeon and a pathologist was associated with significant changes of the CTA-based calcification score (p-value<0.001), using the Ebayes method of the limma package and correcting for the plaque density score protocol (A or B).

## Protein extraction.

Tissue sections were diced and weighed, and approximately 40-80 mg of tissue was taken for three-step protein extraction, as previously described^10^. Briefly, samples were incubated in a NaCl extraction buffer (0.5 M NaCl, 25 mM EDTA, 10 mM Tris pH 7.5, plus protease inhibitors) with slow agitation for 1 h. The NaCl extract (supernatant) was transferred to a new tube and stored at -80 °ׄC for later use. Subsequently, samples were incubated in SDS buffer (0.1% SDS, 25 mM EDTA, and protease inhibitors) for 4 h to release cellular components. Finally, samples were incubated in a guanidine hydrochloride buffer (4 M GuHCl, 50 mM sodium acetate pH 5.8, and protease inhibitors) for 48 h to solubilize long-lived ECM proteins. Protein concentrations were estimated according to the 280 nm absorbance for NaCl and GuHCl extracts, and SDS extracts were quantified using a Pierce BCA protein assay kit according to the manufacturer’s instructions (Pierce BCA Protein Assay Kit, 23225, Thermo Scientific).

## Deglycosylation.

A two-step deglycosylation protocol was employed for GuHCl and NaCl extracts. First, sample pellets were resuspended in deglycosylation buffer (150 mM NaCl, 50 mM sodium acetate, 10 mM EDTA, pH 6.8, plus protease and phosphatase inhibitors) containing the following deglycosylation enzymes: Endo-α-N-acetylgalactosaminidase, α2-3,6,8,9-Neuraminidase, β-1,4-Galactosidase, β-N-Acetylglucosaminidase (all Merck-Millipore Glycoprotein Deglycosylation Kit, 362280), Chondroitinase ABC (Sigma-Aldrich, C3667), Heparinase II (Sigma-Aldrich, H6512), and Endo-β1,4-galactosidase (Sigma-Aldrich, G6920). Samples were incubated at 25 °C for 2 h, followed by 37 °C for 24 h. Second, samples were dried using a SpeedVac (Thermo Scientific, Savant SPD131DDA), reconstituted in ^18^O-labeled water (Taiyo Nippon Sanso, F03-0027) containing PNGase F (Merck-Millipore, 362280), and incubated at 37 °C for 48 h.

## In-solution digestion.

Proteins were denatured using 6 M urea and 2 M thiourea and reduced with 10 mM DTT at 37 °C for 1 h. The samples were then cooled to room temperature before being alkylated using 50 mM iodoacetamide followed by incubation in the dark for 45 min. Pre-chilled (-20 °C) acetone (10x volume) was used to precipitate the samples overnight at -20 °C. Samples were centrifuged at 14,000 x g for 40 min at 4 °C and the supernatant was subsequently discarded. Protein pellets were dried using a SpeedVac, resuspended in 0.1 M triethylammonium bicarbonate (TEAB) buffer, pH 8.2, containing mass spectrometry grade trypsin (Thermo Scientific) (1:50 trypsin: protein), and digested overnight at 37 °C. Trypsin was inhibited by acidification of the samples with a final concentration of 1% trifluoroacetic acid (TFA). Peptide samples were then purified using a 96-well C18 spin plate according to the manufacturer’s instructions (Harvard Apparatus). The dried peptide was reconstituted with 2% acetonitrile (ACN) and 0.05% TFA in water.

## Multiplexed proteomics workflow in the discovery cohort.

For TMT labeling, 5 μg of peptide digests were concentrated using a SpeedVac and resuspended in 10 μl of 50 mM TEAB. A 1 µl aliquot was taken from each sample to create a pool. TMT labeling was carried out according to the manufacturer’s instructions (TMT10plex kit, QK226224, Thermo Scientific). Symptomatic and asymptomatic plaques were equally distributed into the batches of TMT labelling. The pooled sample was measured in the first batch for each batch to normalize the data removing batch effects. After labeling, samples and pools were grouped in equal amounts and dried down using a SpeedVac. Grouped samples were then reconstituted with 2% ACN, 0.05% TFA in water, ready for LC-MS/MS analysis. 5 μg of mixed peptides were separated by a nanoflow LC system (Dionex UltiMate 3000 RSLC nano). Samples were injected onto a nano-trap column (Acclaim PepMap100 C18 Trap, inner diameter 300 µm x length 5 mm, particle size 5 µm, pore size 100 Å), at a flow rate of 25 µL/min for 3 min, using 0.1% formic acid (FA) in water. The following nano-LC gradient was then used at 0.25 µL/min to separate the peptides: 0−3 min, 4% B; 3−10 min, 4–8% B; 10–200 min, 8–30% B; 200–210 min, 30–40% B; 210–215 min, 40–99% B; 215–219.8 min, 99% B; 219.8–220 min, 99–4% B; 220–250 min, 4% B; where A = 0.1% FA in water, B = 80% ACN, 0.1% FA in water. The nano column (EASY-Spray PepMap RSLC C18, 75 µm x 500 mm, 2 μm, 100 Å), set at 40 °C, was connected to an EASY-Spray ion source (Thermo Scientific). Spectra were collected from an Orbitrap mass analyzer (Orbitrap Fusion Lumos Tribrid, Thermo Scientific) using full MS mode (resolution of 120,000 at 400 m/z) over the mass-to-charge (m/z) range 375–1500. Data-dependent MS2 scan was performed using Quadrupole isolation in Top Speed mode using CID activation and ion trap detection in each full MS scan with dynamic exclusion enabled. MS3 was done upon the top 5 MS2 fragment ions with an MS isolation window (m/z) of 0.7, using HCD activation and detected using the Orbitrap at a resolution of 60,000 and scan range (m/z) of 100–500. Three technical replicates were injected for each sample.

Proteome Discoverer software (version 2.1.0.81, Thermo Scientific) was used to search raw data files against the human database (UniProtKB/Swiss-Prot version 2017_01, 20,192 protein entries) using Mascot (version 2.3.01, Matrix Science). The mass tolerance was set at 10 ppm for precursor ions and 0.8 Da for fragment ions. Trypsin was used as the digesting enzyme with up to two missed cleavages being allowed. The in-built TMT10plex static modification was assigned for the detection of TMT labels. Carbamidomethylation of cysteine was chosen as a static modification and oxidation of methionine residues was chosen as a dynamic modification. MS/MS-based peptide and protein identifications were validated with the following filters, a peptide probability of greater than 95.0% (as specified by the Peptide Prophet algorithm), a protein probability of greater than 99.0%, and at least two unique peptides per protein. Data were normalized to the total peptide amount to consider variation in abundances between samples. Scaling was conducted using a control pool sample, correcting for differences in reporter ion abundances due to different numbers of observed peptides.

## Targeted proteomics workflow.

A parallel reaction monitoring (PRM) method was developed on a Q Exactive HF mass spectrometer (Thermo Scientific) with Skyline software (version 4.1, MacCoss Lab Software). Proteotypic peptides of ECM proteins were selected using previous label-free data prioritizing proteins that are changing in at least one comparison in the discovery proteomics analyses. Precursor ions for ECM proteins of interest that were not detected from the label-free analysis were predicted *in silico* using SRM Atlas. Skyline was used to optimize retention times. Proteotypic peptides were scheduled using the retention time obtained from test experiments with the same LC configuration and eluting gradient; retention time windows were set at +/- 4 min. After initial testing, samples were quantified for a final total of 205 peptides for 120 proteins. Samples were injected onto a nano-trap column (Acclaim PepMap100 C18 Trap, same as above), at a flow rate of 25 µL/min for 3 min, using 2% can, 0.1% FA in water. The following LC gradient was then used to separate the peptides at 0.25 µL/min: 0−20 min, 4–8% B; 20−190 min, 8–25% B; 190−210 min, 25−40% B; 210–215 min, 40–99% B; 215–219.8 min, 99% B; 219.8–220 min, 99–4% B; 220–250 min, 4% B; where A = 0.1% FA in water, B = 80% ACN, 0.1% FA in water. The nano column (EASY-Spray PepMap RSLC C18, same as above), set at 40 °C, was connected to an EASY-Spray ion source (Thermo Scientific). Spectra were collected from an Orbitrap mass analyzer (Q Exactive HF, Thermo Scientific) using PRM mode (MS2 resolution of 30,000 at 200 m/z). An isolation window of 2.0 m/z, automatic gain control (AGC) values of 2E5, and a maximum injection time of 60 ms were used. Fragmentation was performed with a normalized collision energy of 28 and MS/MS scans were acquired with a first fixed mass of 110.0 m/z. The identity of a specific peptide was confirmed by the presence of multiple transitions at the same retention time. All peaks were reviewed and integrated manually to ensure accurate peak integration across all samples. Total fragment peak areas were used for quantification. A minimum of three fragments and a signal-to-noise level of 3 to 1 were required for each peptide to qualify as quantifiable. Spearman correlations of peptides belonging to the same protein were computed. In case more than two peptides per protein were detected, peptides were filtered if their correlation with the remaining peptides was less than R = 0.5. In case two peptides per protein were detected, the most abundant peptide was kept even when the correlation was less than R = 0.5. Indexed Retention Time (iRT) peptides were spiked in during the sample preparation and were used for normalization. Final protein abundance was calculated by summing up the quantified peptide abundances. The isolation list is shown in Supplementary material online, Datasets S5.

## Label-free proteomics in the Athero-Express Biobank.

The validation cohort consisted of carotid endarterectomy 200 samples from 200 patients with carotid artery stenosis undergoing carotid endarterectomy. A similar proteomics workflow as the one applied for the discovery was followed but label-free quantification was conducted instead of TMT-labelled quantification. Moreover, a 2-step protein extraction, similar to the previously described extraction protocol but skipping the SDS extraction step, was used. After the protein extraction, 20ug of proteins in GuHCl extracts from each sample were precipitated using 10x volume of ethanol overnight. Next, samples were deglycosylated as described above. After in-solution digestion, the peptide samples were purified using C18 cartridges on a Bravo AssayMAP robotic system (Agilent) according to the manufacturer’s instructions. Regarding the LC-MS/MS label-free analysis protocol, peptides were separated by a nanoflow LC system (Dionex UltiMate 3000 RSLC nano). Samples were injected onto a nano-trap column (Acclaim PepMap100 C18 Trap, inner diameter 300 µm x length 5 mm, particle size 5 µm, pore size 100 Å), at a flow rate of 25 µL/min for 3 min, using 0.1% formic acid (FA) in water. The following nano-LC gradient was then run at 0.25 µL/min to separate the peptides: 0−1 min, 1% B; 1−6 min, 1–6% B; 6–40 min, 6–18% B; 40-70 min, 18-35% B; 70–80 min, 35–45% B; 80–81 min, 45–99% B; 81–89.8 min, 99% B; 89.8–90 min, 99–1% B; 90–120 min, 1% B; where A = 0.1% FA in water, B = 80% ACN, 0.1% FA in water. The nano column (EASY-Spray PepMap RSLC C18, 75 µm x 500 mm, 2 μm, 100 Å), set at 45 °C, was connected to an EASY-Spray ion source (Thermo Scientific). Spectra were collected from an Orbitrap mass analyzer (Q Exactive HF, Thermo Scientific) using full MS mode over the m/z range 350–1600 with a resolution of 60,000 at 200 m/z. Data-dependent MS2 scan was performed using the Top15 method with HCD activation and Orbitrap detection with a resolution of 15,000 in each full MS scan with dynamic exclusion enabled. Thermo Scientific Proteome Discoverer software (version 2.4.1.15) was used to search raw data files against the human database, (UniProtKB/Swiss-Prot version 2021_01, 20,396 protein entries) using Mascot (version 2.6.0, Matrix Science). The mass tolerance was set at 10 ppm for precursor ions and 20 milli mass units (mmu) for fragment ions. Trypsin was used as the digesting enzyme with up to two missed cleavages being allowed. Carbamidomethylation of cysteine was chosen as a static modification. Oxidation of methionine, proline and lysine, and deglycosylation with the presence of O18-water on asparagine were chosen as dynamic modifications. Protein identification FDR confidence was set to high and the minimum number of unique peptides per protein was 2. Only master proteins were shown. The precursor peak area was used for quantification and normalized to total peptide peak area of each sample.

**scRNAseq data integration.**

Analysis on scRNAseq datasets was performed by integrating publicly available single cell (sc) RNAseq datasets of carotid endarterectomies using the harmony integration pipeline^13^. Raw datasets published by Pan et al^44^, Alsaigh et al^45^ and Fernandez et al^46^ were re-processed using the Seurat 4 pipeline^47^.

To estimate the performance of the integration method used we calculated a series of quality metrics which confirmed the ability of the method to remove technical biases maintaining biological variability:

- Normalized Mutual Information (NMI) for cell clustering before and after integration n[Louvain]: 0.6774049
- NMI for cell clustering before and after integration [Leiden]: 0.6863792
- Adjusted Rand Index (ARI) for cell clustering before and after integration [Louvain]: 0.4646822
- ARI for cell clustering before and after integration [Leiden]: 0.465263
- ASW^48^: 0.08549741
- kBET^48^:0.996
- LISI^48^:1.236

CellChat tool^49^ was used to infer and study cell-cell communication patterns using the integrated scRNAseq dataset.

**Spatial RNA-sequencing.**

Visium for formalin-fixed paraffin-embedded (FFPE) spatial gene expression slide and reagent kit was used according to manufacturer instructions (PN: 1000338; 10X Genomics). Each capture area (6.5 x 6.5 mm2) contains 5,000 barcoded spots that are 55 μm in diameter (100 μm center-to-center distance) providing an average resolution of 1 to 10 cells. A 5µm tissue section from one carotid plaque sample was placed onto one capture area of a Visium Spatial Gene Expression slide. Two samples were selected form Asymptomatic male patients for spatial RNA-seq. Deparaffinization, DAPI immunofluorescence staining, image acquisition and decrosslinking were performed as specified in the Visium Spatial Gene Expression for FFPE – Deparaffinization, Decrosslinking, Immunofluorescence Staining & Imaging protocol (CG000410) and performed at the Core Facility Imaging, Medical University of Vienna. Fluorescent images were acquired with an Olympus IX83 microscope equipped with a Hamamathsu Orca FLash camera for fluorescence image capture. and parameters following those specified in the Visium Spatial Gene Expression Reagent Kits for FFPE User Guide sequencing instructions (read 1: 28 cycles; i7 index read: 10 cycles; i5 index read: 10 cycles; and read 2: 50 cycles) yielding 149 million sequenced reads. The FASTQ files and the manually aligned histology images were analyzed with Space Ranger 1.3.1 and the human probeset provided by 10X genomics (Visium Human Transcriptome Probe Set v1.0 GRCh38-2020-A). Visualization of spatial cluster output from Space Ranger as well as differential gene expression analysis to determine cluster-specific up- or downregulated genes was performed on Loupe Browser 6.0.0 (10X Genomics).

## Statistical and bioinformatics analysis.

The proteins retrieved using the GuHCl and NaCl extracts were filtered to keep the ECM and related proteins using ECM protein annotation from the MatrisomeDB^3^ but adding extracellular proteins, such as apolipoproteins, that were deemed important in the context of atherosclerosis. The dataset was further filtered using a method to discriminate between random missing values and values that are consistently missing because of abundances below the limit of detection. Consistent missing values were identified and imputed with zeros when more than 90% missing values were observed in one region of the plaque and less than 10% in the other. Otherwise, proteins with >30% missing values were filtered out. All remaining missing values were imputed with the KNN-Impute method with k equal to 20 (default value). The relative quantities of the proteins were scaled using log2 transformation. The limma package was used to compare between different phenotypes using the Ebayes algorithm and correcting for selected covariates. The p-values were adjusted for multiple testing using the Benjamini-Hochberg method. Spearman correlation was used to correlate the relative expression levels of proteins with clinical variables, imaging, and histology measurements. Network visualizations were conducted using the Cytoscape tool. Enrichment analysis was conducted using the David tool. This analysis included pathway terms from Reactome and Kyoto Encyclopedia of Genes and Genomes (KEGG) and molecular function annotation from Gene Ontology. Survival analysis was conducted using Kaplan-Meier analysis.

The gene expression patterns in different cell types corresponding to the significantly changing proteins were studied using an integrated scRNAseq dataset that combined all three publicly available scRNAseq datasets from human carotid plaques.

ARACNe-AP^50^ software was used to reconstruct correlation networks from the MS proteomics data. This software package is based on conditional mutual information to infer directed weighted correlation links between the nodes using relative expression datasets. 100 bootstraps were used for the reconstruction of the networks and multiple edges and self-loops were filtered. To characterize the edges of the network as inhibition or activation we used the SIREN algorithm^51^. The matrisome network was constructed by combining NaCl and GuHCl extract data. For proteins quantified in both extracts, the extract on which the protein had a higher number of matched spectra was used.

Principal component analysis (PCA) was conducted using the scikit-learn python library version 0.19.2. Scree test was used to retain an adequate number of principal components to maintain at least the 90% variability of the data set. KMEANS algorithm from the python library scikit-learn version 0.19.2 was used for clustering. Inferring the optimal number of clusters was accomplished by experimenting with values from 2 to 20 measuring the Calinski-Harabasz score.

Proteins belonging to the revealed proteomics signatures were considered as potential inputs to classification models to predict the primary endpoint in the 9-year follow-up. An ensemble dimensionality reduction technique was used deploying a multi-objective evolutionary algorithm to a) identify the optimal feature subset to be used as input to Support Vector Machines Classification models and b) optimize the SVM’s parameters values (regularization parameter C and Radial Basis Function parameter gamma). The iterative process of the optimization framework begins by initializing a set of solutions. Each solution consists of i) values for deciding if a feature/proteomic marker will be used as input (values greater than 0.5 force its use) and ii) two values for optimizing the gamma parameter of Radial Basis Functions Kernel and the regularization parameter C of SVM models. The first population of solutions is generated by randomizing values. The optimization goals that were formulated as Fitness Functions were the following:

- Fitness Function 1: Area Under The Receiver Operating Characteristic (ROC) Curve - AUC
- Fitness Function 2: 1/(1+number of support vectors)
- Fitness Function 3: 1/(1+number of selected features)

After the evaluation of the population, the Pareto fronts of non-dominated solutions are calculated, and solutions are assigned a fitness value based on their Pareto front. The Roulette Wheel Selection method is applied to generate a new population of solutions which are then differentiated using the Genetic Algorithms two-point crossover and Gaussian mutation operators. The new population is evaluated again, and this iterative process continues until it converges (the best solution’s performance is less than 5% away from the mean performance of the population for 5 consecutive generations) or reaches the maximum number of generations.

# Major Resources Table

## Data & Code Availability

| **Description** | **Source / Repository** | **Persistent ID / URL** |
| --- | --- | --- |
| Python codes for data preprocessing, statistical and clustering analyses | Github | <https://github.com/konstantinostheofilatos/Vascular_Proteomics-Statistical_Analysis.git> |
| Discovery proteomics raw data from Vienna Cohort | Proteomexchange | PXD030975 |
| Targeted proteomics raw data from Vienna Cohort | Proteomexchange | PXD031052 |
| Raw proteomics data from the Athero-Express Biobank Study | DataverseNL | <https://doi.org/10.34894/4IKE3T> |
| Spatial RNAseq dataset of 2 plaques from Vienna Cohort | Gene Expression Omnibus | GSE241346 |

## Online Supplemental Files

| **File Name** | **Description** |
| --- | --- |
| Supplemental File I | Summary Proteomics and Statistics Results of TMT Proteomics data on SDS extract. |
| Supplemental File II | Summary results and statistics of the TMT Proteomics using the NaCl extract. |
| Supplemental File III | Summary results and statistics of the TMT Proteomics using the GuHCl extract. |
| Supplemental File IV | Differential expression results comparing clusters of spatial single cell RNA-sequencing data from carotid plaques |
| Supplemental File V | Summary details of proteins and peptides quantified using targeted proteomics PRM method |
| Supplemental File VI | Summary results and statistics of the PRM Targeted Proteomics analysis using the GuHCl extract |

# Supplemental Tables

## Table S1. Clinical characteristics of the patient cohort.

|  | **Overall**  **(n=120)** | **Asymptomatic**  **(n=78)** | **Symptomatic**  **(n=42)** | **P-value** |
| --- | --- | --- | --- | --- |
| **Demographics** | | | | |
| Age | 70 (64-74) | 70 (63-73) | 71 (66-75) | 0.21 |
| Sex (male) | 88 (73.3) | 54 (69.0) | 34 (80.1) | 0.20 |
| **Characteristics of carotid artery stenosis** | | | | |
| Grade of stenosis | 90 (85-90) | 90 (90-95) | 90 (80-90) | **0.03** |
| Stenosis grade ≥90% | 67 (55.8) | 47 (60.3) | 20 (47.6) | 0.25 |
| Contralateral stenosis | 34 (28.3) | 19 (24.4) | 15 (35.7) | 0.21 |
| Peak systolic flow velocity, m/s | 3.8 (3 – 4.5) | 3.9 (2.9 -4.5) | 3.6 (3 – 4.4) | 0.61 |
| Plaque morphology | | | | |
| Echogenic | 60 (50) | 43 (55.1) | 17 (40.5) | 0.31 |
| Mixed | 26 (21.7) | 15 (19.2) | 11 (26.2) |  |
| Echolucent | 34 (28.3) | 20 (25.6) | 14 (33.3) |  |
| Histological AHA classification | | | | |
| Type V fibroatheroma | 32 (26.7) | 21 (26.9) | 11 (26.2) | 0.96 |
| Type VI complex lesion | 52 (43.3) | 34 (43.6) | 18 (42.9) |  |
| Type VII calcified lesion | 20 (16.7) | 12 (15.4) | 8 (19.1) |  |
| Type VIII fibrotic lesion | 16 (13.3) | 11 (14.1) | 5 (11.9) |  |
| Calcified (clinical†) | 70 (58.3) | 46 (59.0) | 24 (57.1) | 0.85 |
| **Comorbidities and risk factors** | | | | |
| Transient ischemic attack | 23 (19.2) | 0 (0) | 23 (54.7) | **<0.001** |
| Stroke | 24 (20.0) | 0 (0) | 24 (57.1) | **<0.001** |
| History of stroke/TIA | 23 (19.2) | 14 (18.0) | 9 (21.4) | 0.64 |
| Acute myocardial infarction | 25 (20.8) | 17 (22.0) | 8 (19.1) | 0.82 |
| Coronary artery disease | 42 (35.0) | 24 (30.8) | 18 (42.9) | 0.23 |
| Peripheral artery disease | 49 (40.8) | 37 (47.4) | 12 (28.6) | 0.05 |
| Arterial hypertension | 108 (90.0) | 70 (90.0) | 38 (90.1) | 1 |
| Diabetes mellitus type 2 | 36 (30.0) | 26 (33.0) | 10 (23.8) | 0.30 |
| Obesity (BMI>30) | 28 (23.3) | 20 (25.6) | 8 (19.1) | 0.50 |
| Smoking active | 29 (24.2) | 19 (24.4) | 10 (23.8) | 1 |
| Past smoker | 44 (36.7) | 31 (39.7) | 13 (31.0) | 0.43 |
| Pack-years | 20 (0-45) | 20 (0-45) | 20 (0-40) | 0.47 |
| COPD | 28 (23.3) | 19 (24.4) | 9 (21.4) | 0.82 |
| **Medications** | | | | |
| Ace inhibitors | 48 (40.0) | 32 (41.03) | 16 (38.10) | 0.76 |
| Angiotensin receptor blockers | 41 (34.17) | 24 (30.77) | 17 (40.48) | 0.29 |
| Beta-blockers | 79 (65.83) | 52 (66.67) | 27 (64.29) | 0.79 |
| Diuretics | 34 (28.33) | 20 (25.64) | 14 (33.33) | 0.37 |
| Statins | 97 (80.83) | 59 (75.64) | 38 (90.48) | **0.048** |
| Marcoumar | 6 (5) | 5 (6.41) | 1 (2.38) | 0.33 |
| **Laboratory parameters** | | | | |
| LDL, mg/dL | 83 (68-108) | 80 (65-101) | 91 (79-111) | **0.02** |
| HDL, mg/dL | 49 (41-56) | 50 (41-60) | 45 (38-51) | **0.03** |
| Total cholesterol, mg/dL | 164 (144-193) | 163 (141-191) | 166 (148-194) | 0.51 |
| Triglycerides, mg/dL | 129 (99-197) | 123 (95-191) | 157 (107-198) | 0.27 |
| High-sensitivity CRP, mg/dL | 0.3 (0.1-0.6) | 0.2 (0.1-0.5) | 0.3 (0.2-0.9) | **0.03** |
| Continuous data are shown as median (interquartile range). Dichotomous data are shown as n (%). Mann-Whitney test was used for the statistical comparison of continuous variables between symptomatic and asymptomatic plaques and Fisher’s exact test for the categorical variables. The Chi-square test was used for categorical variables of more than two classes (ultrasound and histology). †Plaques were characterized as calcified or non-calcified based on the classification by two clinicians. All patients of the cohort were Caucasians. AHA, American Heart Association; BMI, body mass index; COPD, chronic obstructive pulmonary disease; CRP, C-reactive protein; HDL, high-density lipoprotein; LDL, low-density lipoprotein; TIA, transient ischemic attack. | | | | |

## Table S2. Clinical characteristics of the discovery cohort in the sex comparison.

|  | **Overall**  **(n=120)** | **Male**  **(n=88)** | **Female**  **(n=32)** | **P-value**‡ |
| --- | --- | --- | --- | --- |
| **Demographics** | | | | |
| Age | 70 (64-74) | 70 (63-73) | 70.5 (66-74) | 0.44 |
| Sex (male) | 88 (73.3) | 88 (100.0) | 0 (0.0) | - |
| **Characteristics of carotid artery stenosis** | | | | |
| Grade of stenosis | 90 (85-90) | 90 (85-90) | 90 (87.5-95) | 0.19 |
| Stenosis grade ≥90% | 67 (55.8) | 44 (50.0) | 23 (71.9) | **0.001** |
| Contralateral stenosis | 34 (28.3) | 27 (30.7) | 7 (21.9) | 0.49 |
| Peak systolic flow velocity, m/s | 3.8 (3 – 4.5) | 3.8 (3- 4.5) | 3.7 (2.8 – 4.5) | 0.93 |
| Plaque Morphology | | | | |
| Echogenic | 58 (48.3) | 38 (43.2) | 20 (62.5) | 0.065 |
| Mixed | 28 (23.3) | 25 (28.4) | 3 (9.4) |  |
| Echolucent | 34 (28.3) | 25 (28.4) | 9 (28.1) |  |
| Histological AHA classification | | | | |
| Type V fibroatheroma | 32 (26.7) | 21 (23.9) | 11 (34.4) | **0.002** |
| Type VI complex lesion | 52 (43.3) | 46 (52.3) | 6 (18.8) |  |
| Type VII calcified lesion | 20 (16.7) | 9 (10.2) | 11 (34.4) |  |
| Type VIII fibrotic lesion | 16 (13.3) | 12 (13.6) | 4 (12.5) |  |
| Calcified (clinical†) | 70 (58.3) | 47 (53.4) | 23 (71.9) | 0.053 |
| **Comorbidities and risk factors** | | | | |
| Transient ischemic attack | 23 (19.2) | 19 (21.6) | 4 (12.5) | 0.43 |
| Stroke | 24 (20.0) | 19 (21.6) | 5 (15.6) | 0.46 |
| History of stroke/TIA | 23 (19.2) | 20 (22.7) | 3 (9.4) | 0.12 |
| Acute myocardial infarction | 25 (20.8) | 22 (25.0) | 3 (9.4) | 0.08 |
| Coronary artery disease | 42 (35.0) | 37 (42.0) | 5 (15.6) | **0.009** |
| Peripheral artery disease | 49 (40.8) | 39 (44.3) | 10 (31.2) | 0.21 |
| Arterial hypertension | 108 (90.0) | 79 (89.8) | 29 (90.6) | 1.00 |
| Diabetes mellitus type 2 | 36 (30.0) | 30 (34.1) | 6 (18.7) | 0.12 |
| Obesity (BMI>30) | 28 (23.3) | 16 (18.2) | 12 (37.5) | 0.05 |
| Smoking active | 29 (24.2) | 21 (23.9) | 8 (25) | 1.00 |
| Past smoker | 44 (36.7) | 37 (42.0) | 7 (21.9) | 0.05 |
| Pack-years | 20 (0-45) | 25 (0-50) | 0 (0-30) | **0.02** |
| COPD | 28 (23.3) | 21 (23.9) | 7 (21.9) | 1.00 |
| **Medications** | | | | |
| Ace inhibitors | 48 (40.0) | 36 (40.91) | 12 (37.5) | 0.74 |
| Angiotensin receptor blockers | 41 (34.17) | 31 (35.23) | 11 (34.38) | 0.93 |
| Beta-blockers | 79 (65.83) | 58 (65.91) | 22 (68.75) | 0.77 |
| Diuretics | 34 (28.33) | 22 (25.00) | 12 (37.50) | 0.18 |
| Statins | 97 (80.83) | 74 (84.09) | 24 (75.00) | 0.26 |
| Marcoumar | 6 (5.00) | 5 (5.68) | 1 (3.13) | 0.57 |
| **Laboratory parameters** | | | | |
| LDL, mg/dL | 83 (67-108) | 84 (68-109) | 80 (56-95) | 0.49 |
| HDL, mg/dL | 49 (41-56) | 46 (39-52) | 57 (48-73) | **<0 .001** |
| Total cholesterol, mg/dL | 164 (143-193) | 163 (139-195) | 170 (149-190) | 0.43 |
| Triglycerides, mg/dL | 128 (98-197) | 141 (96-215) | 118.5 (102-162) | 0.16 |
| High-sensitivity CRP, mg/dL | 0.3 (0.1-0.6) | 0.3 (0.1-0.6) | 0.4 (0.1-0.7) | 0.34 |
| Continuous data are shown as median (interquartile range). Dichotomous data are shown as n (%). Mann-Whitney test was used for the statistical comparison of continuous variables between male and female plaques and Fisher’s exact test for the categorical variables. The Chi-square test was used for categorical variables of more than two classes (ultrasound and histology). †Plaques were characterized as calcified or non-calcified based on the classification by two clinicians. AHA, American Heart Association; BMI, body mass index; COPD, chronic obstructive pulmonary disease; CRP, C-reactive protein; HDL, high-density lipoprotein; LDL, low-density lipoprotein; TIA, transient ischemic attack. | | | | |

## Table S3. Clinical characteristics of the validation cohort (Athero-Express) in the sex comparison.

|  | **Overall**  **(n=200)** | **Male**  **(n=149)** | **Female**  **(n=51)** | **P-value**‡ |
| --- | --- | --- | --- | --- |
| **Demographics** | | | | |
| Age | 70 (62-76) | 71 (62-76) | 70 (62-75) | 0.322 |
| Sex (male) | 149 (74.5) | 149 (100.0) | 0 (0.0) | - |
| **Composite Endpoint and Plaque Vulnerability** | | | | |
| Primary Endpoint in three years | 51 (25.5) | 44 (29.5) | 7 (13.7) | **0.025** |
| Plaque Vulnerability Index (0-5) | 2 (2-3) | 3 (2-3) | 2 (1-3) | **0.003** |
| **Comorbidities and risk factors** | | | | |
| Stroke | 30 (15.0) | 25 (16.8) | 5 (9.8) | 0.229 |
| Coronary artery disease | 68 (34.0) | 53 (35.6) | 15 (29.4) | 0.123 |
| Arterial hypertension | 139 (69.5) | 101 (67.8) | 38 (74.5) | 0.368 |
| Diabetes mellitus type 2 | 46 (23.0) | 38 (25.5) | 8 (15.7) | 0.151 |
| Obesity (BMI>30) | 37 (18.5) | 26 (17.4) | 11 (21.6) | 0.428 |
| Smoking active | 71 (35.5) | 50 (33.6) | 21 (41.2) | 0.326 |
| Past smoker | 94 (47.0) | 76 (51) | 18 (35.3) | 0.052 |
| **Medications** | | | | |
| Statins | 157 (78.5) | 117 (78.5) | 40 (78.4) | 1.000 |
| **Laboratory parameters** | | | | |
| LDL, mg/dL | 105 (74-128) | 99 (75-129) | 102 (65-125) | 0.582 |
| HDL, mg/dL | 43 (32-52) | 40 (32-50) | 48 (37-60) | **0.018** |
| Total cholesterol, mg/dL | 174 (142-203) | 171 (144-200) | 185 (139-216) | 0.453 |
| Triglycerides, mg/dL | 129 (87-150) | 112 (87-150) | 124 (88-165) | 0.576 |
| Continuous and ordinal variables are shown as median (interquartile range). Dichotomous variables are shown as n (%). Mann-Whitney test was used for the statistical comparison of continuous and ordinal variables between male and female plaques and Fisher’s exact test for the categorical variables. The Chi-square test was used for categorical variables of more than two classes (ultrasound and histology). †Plaques were characterized as calcified or non-calcified based on the classification by two clinicians. AHA, American Heart Association; BMI, body mass index; COPD, chronic obstructive pulmonary disease; CRP, C-reactive protein; HDL, high-density lipoprotein; LDL, low-density lipoprotein; TIA, transient ischemic attack. | | | | |

## Table S4. Machine learning analysis for the prediction of the follow-up primary endpoint.

| **Dataset** | **Method** | **AUC**† | **Precision** | **Recall** |
| --- | --- | --- | --- | --- |
| Discovery Cohort | Ultrasound  (Echolucent and Mixed vs Echogenic) | 46.6% | 26.2% | 40.7% |
|  | Histology  (Complex and Calcified vs Fibroatheroma and Fibrotic) | 50.0% | 39.0% | 61.7% |
|  | Proteomic Clustering  (Clusters 1 and 2 vs Clusters 3 and 4) | 60.4% | 45.1% | 62.2% |
|  | Tissue Machine Learning Signature by MOEA/SVM:  CNN1, PROC, SERPH, CSPG2 * | 75.0% | 63.4% | 74.2% |
| Validation Cohort –  Athero-Express | Plaque Vulnerability Index  (Categories 0, 1 and 2 vs Categories 3, 4 and 5) | 51.0% | 25.0% | 50.0% |
|  | Machine Learning Signature by MOEA/SVM:  CNN1, PROC, SERPH, CSPG2 | 67.5% | 43.1% | 86.3% |
| CNN1 - calponin, PROC - vitamin-K dependent protein C, SERPH - serpin H1, and CSPG2- versican. MOEA/SVM: Hybrid method combining Multi-Objective Evolutionary Optimisation Algorithm and Support Vector Machines. *Performance metrics for the machine learning biosignature in the discovery cohort were measured using 10-fold cross-validation. †Area Under the Curve (AUC) of the receiver operating characteristic curve. | | | | |
