# Supplementary material for: Proteomic Atlas of Atherosclerosis: The Contribution of Proteoglycans to Sex Differences, Plaque Phenotypes, and Outcomes
Source: Circ Res. 2023 Aug 30;133(7):542–58. doi: 10.1161/CIRCRESAHA.123.322590 (PMC10498884; doi:10.1161/CIRCRESAHA.123.322590)
Supplement: Supplementary file 9 [file res-133-542-s009.pdf]

Major Resources Table

Data & Code Availability

Description	Source / Repository	Persistent ID / URL
Python codes for data preprocessing, statistical and clustering analyses	Github	https://github.com/konstantinostheofilatos/Vascular_Proteomics-Statistical_Analysis.git
Discovery proteomics raw data from Vienna Cohort	Proteomexchange	PXD030975
Targeted proteomics raw data from Vienna Cohort	Proteomexchange	PXD031052
Raw proteomics data from the Athero-Express Biobank Study	DataverseNL	https://doi.org/10.34894/4IKE3T

Online Supplemental Files

File Name	Description
Supplemental File I	Summary Proteomics and Statistics Results of TMT Proteomics data on SDS extract.
Supplemental File II	Summary results and statistics of the TMT Proteomics using the NaCl extract.
Supplemental File III	Summary results and statistics of the TMT Proteomics using the GuHCl extract.
Supplemental File IV	Differential expression results comparing clusters of spatiotemporal single cell RNA-sequencing data from carotid plaques
Supplemental File V	Summary details of proteins and peptides quantified using targeted proteomics PRM method
Supplemental File VI	Summary results and statistics of the PRM Targeted Proteomics analysis using the GuHCl extract
